# Lytic gene expression in the temperate bacteriophage GIL01 is activated by a phage-encoded LexA homologue

**DOI:** 10.1093/nar/gky646

**Published:** 2018-07-24

**Authors:** Nadine Fornelos, Douglas F Browning, Anja Pavlin, Zdravko Podlesek, Vesna Hodnik, Margarita Salas, Matej Butala

**Affiliations:** 1 Instituto de Biología Molecular ‘Eladio Viñuela’ (CSIC), Centro de Biología Molecular ‘Severo Ochoa’ (CSIC-Universidad Autónoma de Madrid), Cantoblanco, 28049 Madrid, Spain; 2 Institute of Microbiology and Infection, School of Biosciences, University of Birmingham, Birmingham B15 2TT, UK; 3 Department of Biology, Biotechnical Faculty, University of Ljubljana, 1000 Ljubljana, Slovenia

## Abstract

The GIL01 bacteriophage is a temperate phage that infects the insect pathogen *Bacillus thuringiensis*. During the lytic cycle, phage gene transcription is initiated from three promoters: *P1* and *P2*, which control the expression of the early phage genes involved in genome replication and *P3*, which controls the expression of the late genes responsible for virion maturation and host lysis. Unlike most temperate phages, GIL01 lysogeny is not maintained by a dedicated phage repressor but rather by the host’s regulator of the SOS response, LexA. Previously we showed that the lytic cycle was induced by DNA damage and that LexA, in conjunction with phage-encoded protein gp7, repressed *P1*. Here we examine the lytic/lysogenic switch in more detail and show that *P3* is also repressed by a LexA–gp7 complex, binding to tandem LexA boxes within the promoter. We also demonstrate that expression from *P3* is considerably delayed after DNA damage, requiring the phage-encoded DNA binding protein, gp6. Surprisingly, gp6 is homologous to LexA itself and, thus, is a rare example of a LexA homologue directly activating transcription. We propose that the interplay between these two LexA family members, with opposing functions, ensures the timely expression of GIL01 phage late genes.

## INTRODUCTION

Bacteriophages fall into two major types, depending on their developmental programmes on infecting a bacterial host cell. Lytic phages initiate the lytic cycle immediately after infecting a cell, leading to host cell lysis and death, whereas temperate phages (also referred to as lysogenic phages) can immediately engage in the lytic cycle or lie dormant inside the host for many generations. Dormant phages retain the ability to initiate the lytic cycle, usually in response to an external trigger that activates the phage regulatory machinery, e.g. DNA damage of the host’s chromosome. The majority of known temperate phages insert their genomes into the host chromosome during lysogeny, where they are replicated at each cell division and are faithfully transmitted to progeny cells, whilst other lysogenic phage exist as an autonomously replicating entity. To date lysogeny has only been thoroughly studied in detail for a handful of phages, and yet it is clear that temperate bacteriophages have a great impact on bacterial evolution, leading to the rearrangement of bacterial genomes and acquisition of potent virulence determinants (1).

**Figure 1.**
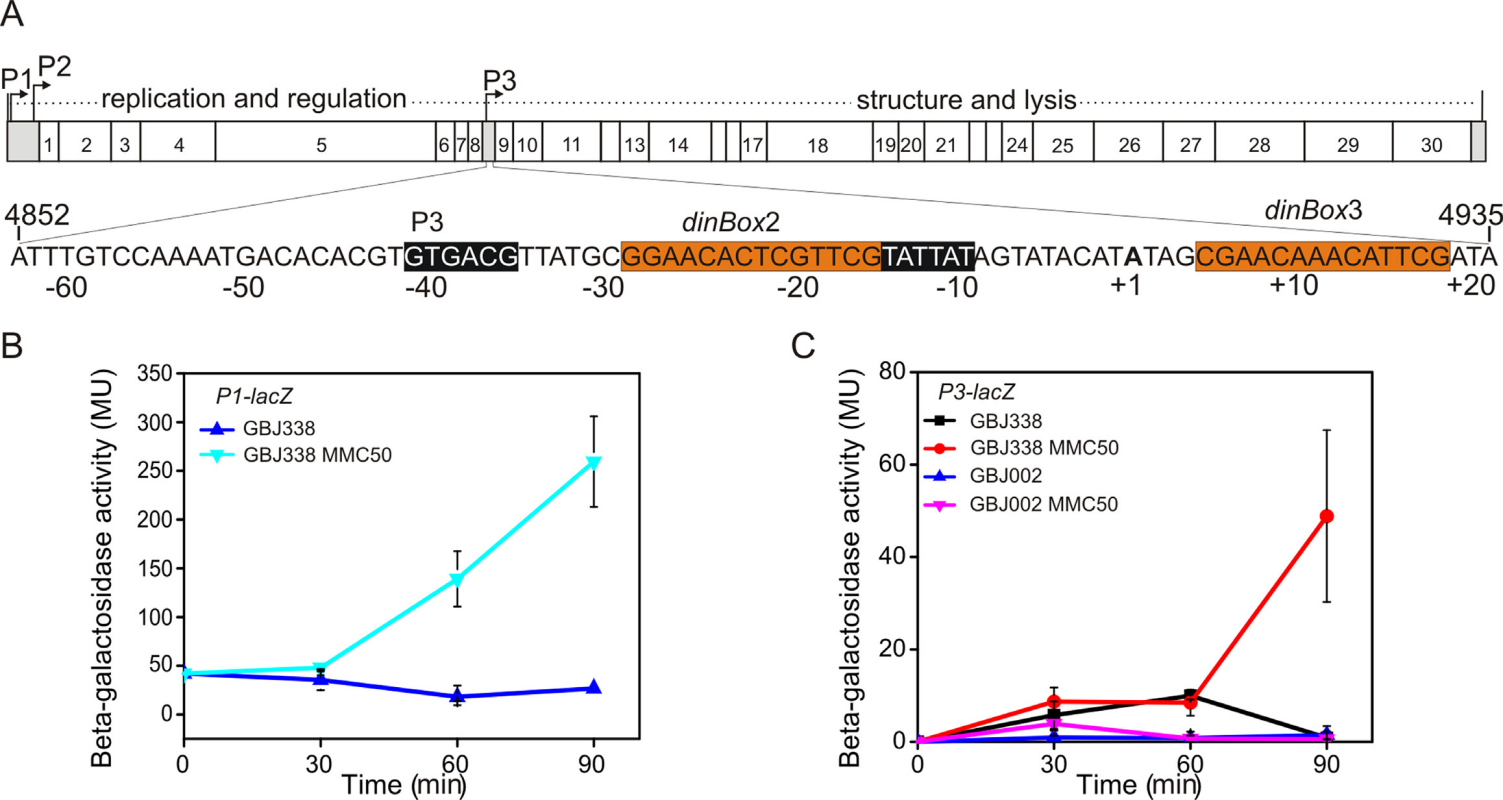
An uncharacterized phage-encoded factor controls the activity of the *P3* lytic promoter. (**A**) A schematic representation of the GIL01 genome with the *P3* promoter sequence enlarged below. The putative −35 and −10 core promoter elements are highlighted in black, LexA-binding sites are highlighted in orange and the transcription start site (+1) is shown in bold. The GIL01 genome sequence coordinates are indicated. The figure also shows measured β-galactosidase activities of (**B**) the *Bacillus thuringiensis* GIL01 lysogenic strain, GBJ338, carrying a promoter *P1–lacZ* fusion and (**C**) GBJ338 and its GIL01-cured derivative, GBJ002, each carrying a promoter *P3*–*lacZ* fusion. Where indicated, a sub-inhibitory concentration of mitomycin C (MMC50, 50 ng/ml) was added at time 0. Each value represents the mean ± standard deviation of three independent measurements.

The Tectiviruses are bacteriophages that share structural similarities with several eukaryotic and archaeal viruses (2,3). On account of these similarities, it has been suggested that Tectiviruses have played an important role in early eukaryotic evolution, being the precursors of Polintons (large eukaryotic DNA transposons), which are thought to have evolved into most of the large double-stranded DNA eukaryotic viruses observed today (3). Tectiviral bacteriophages are characterized by a linear genome capped at both extremities by covalently bound terminal proteins that act as primers during DNA replication. In the mature phage particle, the linear genome is contained inside a protein-rich lipid membrane that is surrounded by a rigid protein capsid icosahedron with flexible spikes (4). Most of the Tectiviruses characterized to date are lytic phages infecting enterobacteria (e.g.*Escherichia coli* and *Salmonella enterica*) and their genomes are highly similar, despite each phage originating from distinct geographical locations (5). However, recent genomic analysis has indicated that the Tectiviridae are widely distributed, with Tectivirus-related elements being present in the genomes of *Streptococcus, Exiguobacterium, Clostridium, Brevibacillus* and *Rhodococcus* species, for example (6–8). Temperate Tectiviruses have so far only been shown to infect the *Bacillus cereus* group, of which the opportunistic pathogens *B. cereus, Bacillus thuringiensis* and *Bacillus anthracis* are the most notable members (9). The Tectivirus phage GIL01 infects the insect pathogen *B. thuringiensis* and can establish a stable lysogenic state inside the cell, where it resides as a 15-kbp extrachromosomal linear replicon (Figure 1A) (10). Lysogeny is stably perpetuated over generations until the host cell experiences genomic stress and DNA damage, and this is the inducer for GIL01 resurrection, with DNA replication, particle formation and host cell lysis being initiated (10,11).

For GIL01, the decision to enter the lytic or lysogenic life cycle is controlled by three promoter regions (*P1, P2* and *P3*), which likely guide the transcription of all predicted 30 phage genes in the same direction (Figure 1A). The tandem *P1* and *P2* promoters, at the extreme left of the GIL01 genome, control the expression of the phage early genes involved in genome replication and transcription regulation (Figure 1A) (11,12). In contrast to most temperate phages, which encode their own repressor of the lytic cycle, phage GIL01 exploits the host’s LexA repressor to regulate its lifestyle choices (11–13). LexA is the repressor of the SOS response to DNA damage in bacteria and, in Gram-positive bacteria, recognizes and binds to palindromic sequences at promoters controlling the expression of DNA repair genes and of several other genes involved in bacterial virulence and evolution (14,15). DNA damage, such as that inflicted by genotoxic agents or caused by spontaneous DNA breaks during replication, triggers LexA self-cleavage and the expression of the SOS regulon (13). In phage GIL01, host LexA binds downstream of *P1* to a site named *dinBox*1, repressing the expression of genes involved in phage genome replication. Previously, we demonstrated that the small GIL01-encoded gp7 polypeptide formed a complex with LexA, and enabled it to bind to a second low-affinity LexA box, *dinBox*1b (12,13). In doing this gp7 stabilized LexA binding to the promoter DNA, without binding the DNA itself, and prevented LexA auto-cleavage and premature phage reactivation. The internal lytic promoter *P3* controls the expression of the GIL01 capsid and lytic genes (11,16). Like *P1, P3* appears to be regulated by host LexA *in vivo*, with LexA potentially binding to two proposed sites, *dinBox*2 and *dinBox*3 (Figure 1A) (11,12). Here, we show that LexA binds these sequences and that gp7 also forms a complex with LexA, to alter repressor binding to these two sites. We also show that DNA damage alone is not sufficient to trigger expression from *P3* and that *P3* requires the expression of a second small GIL01-encoded protein, gp6. Using *in vitro* and *in vivo* approaches, we show that gp6 directly activates transcription from *P3* and identify its DNA binding site within the *P3* promoter. As gp6 is a truncated homologue of LexA, this is the first example of a promoter controlled by diverse LexA family members, with opposing functions. We, therefore, propose that the dependence of *P3* on both gp6 and LexA is a mechanism that ensures that the expression of the GIL01 late genes, encoding capsid and lytic proteins, is delayed and only occurs on the accumulation of gp6 protein after sustained DNA damage.

## MATERIALS AND METHODS

### Bacterial strains and culture conditions

All strains and plasmids used in the study are presented in Supplementary Table S1. The *B. thuringiensis* serovar *israelensis* GIL01-lysogenic host, GBJ338, and the GIL01-cured strain, GBJ002 (11,17), were grown aerobically in L-broth at 30°C. Derivatives of the *lacZ* reporter plasmid pHT304-18Z, pDin1, carrying the *P1*–*lacZ* promoter fusion and pDin3, carrying the *P3*–*lacZ* promoter fusion (11), were maintained in *B. thuringiensis* by supplementing media with 25 μg/ml erythromycin. To express gp6 in *B. thuringiensis*, GBJ002 cells, carrying plasmid pDin3 and the gp6 overexpression plasmids pDG6 or pDG6 K38A, were grown in L-broth supplemented with 25 μg/ml erythromycin and 25 μg/ml kanamycin, and expression was induced with 0.1 mM isopropyl β-d-1-thiogalactopyranoside (IPTG) 1 h after sub-culturing cells into fresh L-broth (1:100). To induce DNA damage, 50 ng/ml mitomycin C was added 3 h after inoculation to exponentially growing *B. thuringiensis* cultures. At selected time points, 20 μl of each culture was assayed for β-galactosidase activity as described in (11). Plasmid pSR/GIL01 *P3* and pET8c derivatives pgp6 and pgp6 K38A were maintained in *E. coli* cells by the addition of 100 μg/ml ampicillin to cultures.

### Plasmid construction

ORF6 from phage GIL01 (coordinates 4350 through 4550; GenBank accession number AJ536073) was polymerase chain reaction (PCR) amplified, using Vent DNA polymerase (NEB) with primers gp6_u and gp6_d flanked by BamHI and MluI sites (oligonucleotides are listed in Supplementary Table S2). Purified PCR product was digested with BamHI and MluI and cloned into expression vector pET8c (Novagen), generating plasmid pgp6, which was used to overexpress gp6 protein carrying an N-terminal hexahistidine-tag (His_6_) and a thrombin cleavage site. To create the pgp6 K38A plasmid, which overexpresses the gp6 K38A mutant protein, substitutions were introduced in plasmid pgp6 using the QuikChange site-directed mutagenesis kit (Stratagene) with primer pair K38A_u and K38A_d and pgp6 as a template, according to manufacturer’s instructions. To construct plasmid pDG6 for IPTG-induced expression of gp6 in *B. thuringiensis*, ORF6 was PCR amplified using primers gp6BT_u and gp6BT_d flanked by XbaI and SphI sites and cloned into XbaI- and SphI-digested pDG148 plasmid. To construct plasmid pDG6 K38A, the pDG6 plasmid was amplified using Phusion high-fidelity DNA polymerase (NEB) with a pair of complementary primers carrying the K38A mutation, K38A_u and K38A_d. Amplification was carried out for 16 cycles of 95°C for 30 s, 53°C for 60 s and 72°C for 180 s. The resulting DNA was DpnI-treated to digest away the methylated template DNA and the newly synthesized pDG6 K38A mutant DNA was propagated in the *E. coli dam*^−^ strain JW3350 before being electroporated into *B. thuringiensis* GBJ002 cells. The GIL01 *P3* promoter fragment, which carries the GIL01 *P3* promoter, was amplified using primers GILp3 (up) and GILp3 (down) with GIL01 DNA as template. Purified PCR was restricted with EcoRI and HindIII and cloned into plasmid pSR to generate pSR/GIL01 *P3*. Plasmid DNA was then used as template for *in vitro* transcription assays and as a source of GIL01 *P3* promoter fragment DNA for both electrophoretic mobility shift assay (EMSA) and DNase I footprinting analysis.

### Purified proteins

Purified *Bacillus subtilis* RNA polymerase holoenzyme, complexed with the SigA sigma factor in 50 mM Tris–HCl (pH 8.0), 100 mM NaCl, 3 mM β-mercaptoethanol and 50% glycerol (18), was kindly donated by Dr Libor Krasny (Chech Academy of Sciences, Chech Republic). Note that the corresponding RNA polymerase subunits from *B. subtilis* and *B. thuringiensis* are highly similar and that *B. subtilis* RNA polymerase holoenzyme can transcribe *B. thuringiensis* genes (19). Recombinant *B. thuringiensis* LexA (coordinates 3 624 082 through 3 624 714; GenBank accession number: CP_001186) and GIL01 gp7 (coordinates 4564 through 4716; GenBank accession number AJ536073), carrying N-terminal His_6_-tags, were overexpressed in *E. coli* strain M15 from plasmids pQELexA and pQE7 and purified as previously described in (12). To overexpress recombinant gp6 and gp6 K38A proteins, carrying N-terminal His_6_-tags and the thrombin cleavage site, BL21(DE3) pLysE cells, carrying either pgp6 or the pgp6 K38A plasmid, were grown aerobically at 37°C in 500 ml of L-broth supplemented with ampicillin (100 μg/ml) and chloramphenicol (25 μg/ml) to an optical density at 600 nm (OD_600_) of 0.5. The culture was cooled to 20°C and 0.6 mM IPTG was added to the culture. After 4 h of growth at 20°C, with shaking at 180 rpm, cells were harvested and the N-terminally His_6_-tagged gp6 and His_6_-gp6 K38A were affinity purified by Ni-chelate chromatography (Qiagen), with columns pre-equilibrated, washed and proteins eluted with buffer A (50 mM NaH_2_PO_4_, 0.3 M NaCl, pH 8.0) containing 10, 20 or 250 mM imidazole, respectively. A Slide-A-Lyzer dialysis cassette with 3.5-kDa molecular weight cutoff (Thermo Scientific) was used to exchange the elution buffer directly into 20 mM Tris–HCl (pH 7.3), 140 mM NaCl, 5 mM ethylenediaminetetraacetic acid (EDTA), 2 μM dithiothreitol (DTT) and stored at −80°C. For simplicity, all the tagged proteins will be referred to in future without reference to the His_6_ moiety (e.g. LexA, gp6, gp6 K38A and gp7). The concentration of the recombinant LexA, gp7, gp6 and gp6 K38A proteins was determined using NanoDrop1000 (Thermo Scientific) and the extinction coefficients at 280 nm used were 7450, 2980, 4470 or 4470 M^−1^ cm^−1^, respectively.

### EMSA analysis

For EMSA experiments, involving *P3* promoter fragments that carried *dinBox*2 and/or *dinBox*3 in various combinations, DNA probes were generated using PCR with oligonucleotides EMSA7 to EMSA10. Products were purified, P^32^ end-labelled and the interaction of LexA and complexed gp7 was assayed as described in (12). EMSA experiments investigating the interaction of LexA, gp6 and gp6 K38A used purified AatII–HindIII GIL01 *P3* promoter fragment, obtained from pSR/GIL01 *P3*. Purified DNA fragment was again P^32^ end-labelled and EMSA analysis was carried out as in (20). Data from experiments were visualized and quantified using a Bio-Rad Molecular Imager FX and Quantity One software.

### DNase I footprint analysis

To assay the interaction of LexA and gp7 with the GIL01 *P3* promoter region using DNase I footprinting, EMSA7 and EMSA10 oligonuleotides, flanking *P3*, were used to amplify the 200 bp probe and the upper DNA strand was P^32^ end-labelled. DNase I footprinting experiments were performed as detailed in (12). For experiments investigating the interaction of LexA and gp6, purified AatII–HindIII GIL01 *P3* promoter fragment was P^32^ end-labelled on the bottom strand and DNase I footprinting was performed as in (21). Samples were analyzed by gel electrophoresis using denaturing polyacrylamide gels, containing 1× Tris-borate-EDTA (TBE), and were calibrated with Maxam-Gilbert ‘G+A’ sequencing reactions of the labelled fragment. Gels were analyzed and quantified using a Bio-Rad Molecular Imager FX and Quantity One software (Bio-Rad).

### *In vitro* transcription assays

*In vitro* transcription assays were carried out as detailed in (22) using purified gp6 and LexA proteins, purified *B. subtilis* RNA polymerase and pSR/GIL01 *P3* plasmid as template. Samples were loaded onto a denaturing polyacrylamide gel, containing 1× TBE, which was calibrated using P^32^ end-labelled 100 bp ladder (NEB). Gels were analyzed using a Bio-Rad Molecular Imager FX and Quantity One software (Bio-Rad).

### Surface plasmon resonance assays

The surface plasmon resonance (SPR) measurements were performed on a Biacore T100 (GE Healthcare) at 25°C. Approximately 30 response units (RU) of 3′-biotynilated S1 primer was immobilized on the flow cells of a streptavidin chip previously equilibrated in running buffer (25 mM Tris–HCl (pH 7.4), 140 mM NaCl, 5 mM EDTA, 2 mM DTT, 0.1 mg/ml bovine serum albumin, 0.005% surfactant P20). To prepare double-stranded DNA fragments carrying the putative LexA or gp6 target sequences within the *P3* promoter, complementary primers (denoted as primer name_u and primer name_d) (Supplementary Table S1) were annealed using a temperature gradient as described in (12). The resulting DNA probes were 36–89 bp long and carried a 15 nt overhang complementary to the SPR chip-immobilized S1 primer at their 5’-end. A total of 30–100 RU of each DNA probe was immobilized onto each flow cell 2 at 2 μl/min, as detailed in the figure legends. The interaction between chip-immobilized DNAs and gp6, gp7, and LexA in different combinations was studied by injecting solutions at the desired protein concentration in running buffer at a rate of 100 μl/min. Regeneration of the sensor surface was performed with 50 mM NaOH for 10 s. SPR experiments were performed at the Infrastructural Centre for Analysis of Molecular Interactions at the Department of Biology, University of Ljubljana.

### Bioinformatics

Clustal Omega (23) was used to align nucleotide and amino acid sequences and figures were created using BoxShade (https://embnet.vital-it.ch/software/BOX_form.html). To construct the homology model of GIL01 gp6, we used SWISS-MODEL (24) and the crystal structure of the *Thermotoga maritima* LexA repressor as a template (PDB ID: 3k2z). Visualization and superposition of gp6 model and the crystal structure of the DNA bound *E. coli* LexA repressor (PDB ID: 3jso) (25) were performed using the Visual Molecular Dynamics program (26).

## RESULTS

### The *P3* lytic promoter is repressed by a LexA–gp7 complex and requires a phage-encoded factor for induction

Previously, we investigated the regulation of the GIL01 *P1* promoter and demonstrated that expression from this promoter was induced by DNA damage and that *P1* was co-ordinately regulated by LexA and the small GIL01 phage-encoded protein, gp7 (Figure 1A) (11). To investigate the regulation of the GIL01 *P3* promoter, a 227-bp DNA fragment, carrying the *P3* promoter region, was cloned into the low-copy-number *lacZ* reporter plasmid pHT304-18Z and β-galactosidase expression was determined in the *B. thuringiensis* strain GBJ338 (a GIL01 lysogen) after mitomycin C-induced DNA damage. Expression from *P3* was compared to a *P1*–*lacZ* promoter fusion in the same strain background over time (12). Results in Figure 1 show that while *P1* was induced in the lysogenic strain after 30 min of exposure to mitomycin C (Figure 1B), *P3* expression was also induced by DNA damage but induction was delayed by 1 h (Figure 1C). Interestingly, when *P3* expression was examined in the *B. thuringiensis* strain GBJ002, which has been cured of GIL01, mitomycin C-induced DNA damage failed to stimulate expression from *P3*, suggesting that phage-specific factors might also control the *P3* promoter (Figure 1C).

**Figure 2.**
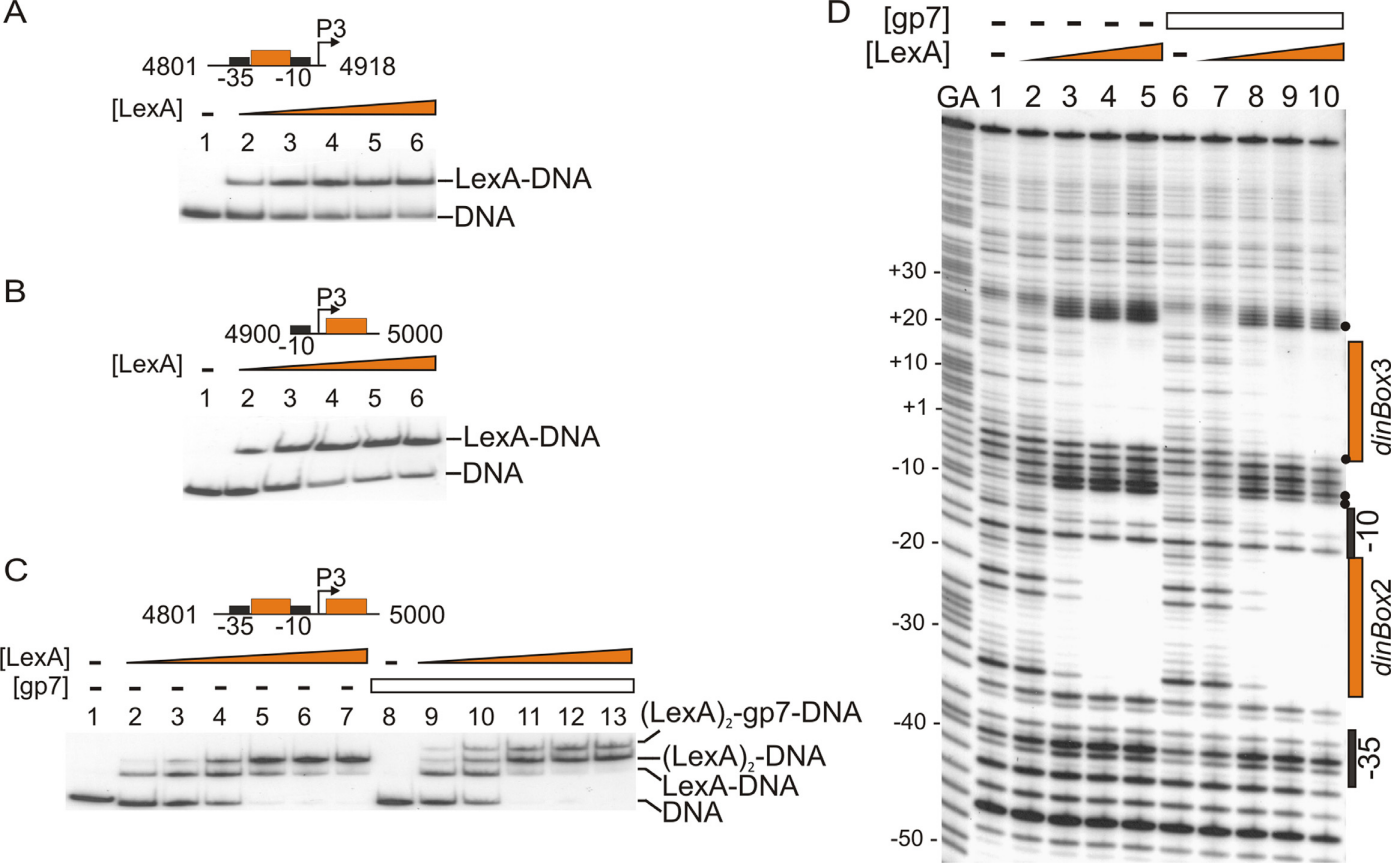
Phage-encoded gp7 protein modulates the binding of LexA to the *P3* SOS operator sequences. (**A–C**) The binding of purified LexA and gp7 protein to various P^32^ end-labelled *P3* promoter fragments was assayed by EMSA. Above each gel is a schematic representation of the *P3* promoter fragment used. The corresponding GIL01 genome coordinates are also given, with LexA protein binding sites shown as orange boxes and the −10 and −35 promoter elements boxed in black. The concentration of LexA in panels (A) and (B) in lanes 2–6 was 0.17, 0.32, 0.66, 1.33 and 13.28 nM, respectively. The concentration of LexA in panel (C) in lanes 2–7 was 0.08, 0.17, 0.32, 0.66, 1.33 and 13.28 nM, and in lanes 9–13 was 0.17, 0.32, 0.66, 1.33 and 13.28 nM, respectively. The concentration of gp7 in lanes 8–13 in panel (C) was 17 nM. The location of free DNA and the position of various LexA–gp7–DNA complexes are indicated. (**D**) DNase I footprint analysis showing the effect that gp7 has on LexA binding to its tandem operators. A DNA fragment extending from position 4801 to 5000 relative to the GIL01 genome sequence (as in panel C) was ^32^P-labelled, incubated with either LexA or LexA and gp7, and digested with DNase I. The gel was calibrated using Maxam–Gilbert G+A sequencing reactions of the labelled fragment (designated as GA), and selected positions are indicated. The concentration of LexA in lanes 1–5 and 6–10 was 0, 20, 80, 320 and 480 nM, respectively. In lanes 6–10, 340 nM gp7 was also added to the reaction mixtures. LexA operators and the promoter elements are indicated to the right of the gel and LexA-induced hypersensitive sites are indicated by black dots.

Inspection of the DNA sequence downstream of the *P3* −35 promoter element revealed two putative targets for the LexA repressor, which were previously designated *dinBox*2 and *dinBox*3 (Figure 1A). As this suggested that LexA might directly control *P3* in response to DNA damage, we investigated whether LexA bound to these targets using EMSA with purified LexA and radiolabelled DNA fragments that carried either the *dinBox*2 and *dinBox*3 LexA sites individually or in combination. Results in Figure 2 reveal that LexA could bind to all DNA fragments tested, suggesting that LexA can bind independently to each LexA operator (Figure 2A and B) and occupy both sites simultaneously (Figure 2C). DNase I footprinting analysis of the radiolabelled *P3* promoter region, using purified LexA protein, also confirmed that LexA bound to these sites (Figure 2D). Additional experiments, using SPR analysis, indicated that the LexA repressor bound to these sites in a concentration-dependent manner and that LexA DNA binding was particularly stable (Supplementary Figure S1), implying that *dinBox*2 and *dinBox*3 are high-affinity operators, which is the characteristic of SOS genes expressed late in the DNA damage response (27). Furthermore, the mutation of these LexA boxes completely abolished LexA binding in SPR analysis, confirming that LexA specifically recognizes *dinBox*2 and *dinBox*3 (Supplementary Figure S1D). Thus, we conclude that LexA binds specifically to two high-affinity sites within the GIL01 *P3* promoter, *dinBox*2 and *dinBox*3.

As we previously showed that the phage-encoded gp7 protein forms a complex with LexA and enables the repressor to occupy sites at the *P1* promoter to establish the lysogenic cycle (11,12), we examined if gp7 could also modulate LexA binding at the *P3* promoter. This was initially investigated using EMSA analysis, which demonstrated that gp7 protein could bind to this LexA–DNA complex by generating a shift of higher molecular weight (Figure 2C). The association of gp7 with the LexA–DNA complex was also confirmed using SPR analysis (Supplementary Figure S2). Note that gp7 alone does not bind to the DNA (Figure 2C and Supplementary Figure S2) (12). DNase I footprint analysis was also used to study LexA–gp7 binding to the *P3* promoter and indicated that gp7 altered the interaction of LexA with the *P3* promoter region, as the DNase I hypersensitive sites observed immediately downstream of the −10 promoter element were altered (Figure 2D). This is indicative of a local narrowing of the DNA minor groove and stabilization of the DNA helix by LexA in conjunction with gp7 (28,29). Thus, we conclude that the phage-encoded protein gp7 modulates the binding of LexA to *dinBox*2 and *dinBox*3 by altering the architecture of the LexA–DNA complex.

As *dinBox*2 and *dinBox*3 are separated by approximately two DNA helix turns, it is possible that this sequence organization is required for gp7 to interact with both dimers of LexA, when bound to the DNA. To investigate this, we inserted either five or ten nucleotides between the two LexA operators, separating LexA repressor molecules by a half- or one-DNA helical turn, respectively, and examined the binding of LexA and gp7 using SPR. Results showed that gp7 protein was able to complex with LexA bound to each of the DNA fragments tested, regardless of operator spacing (Supplementary Figure S3). Thus, our data suggest that at the *P3* promoter gp7 acts on individual LexA repressor dimers.

### The small phage protein gp6 activates expression from the *P3* lytic promoter

**Figure 3.**
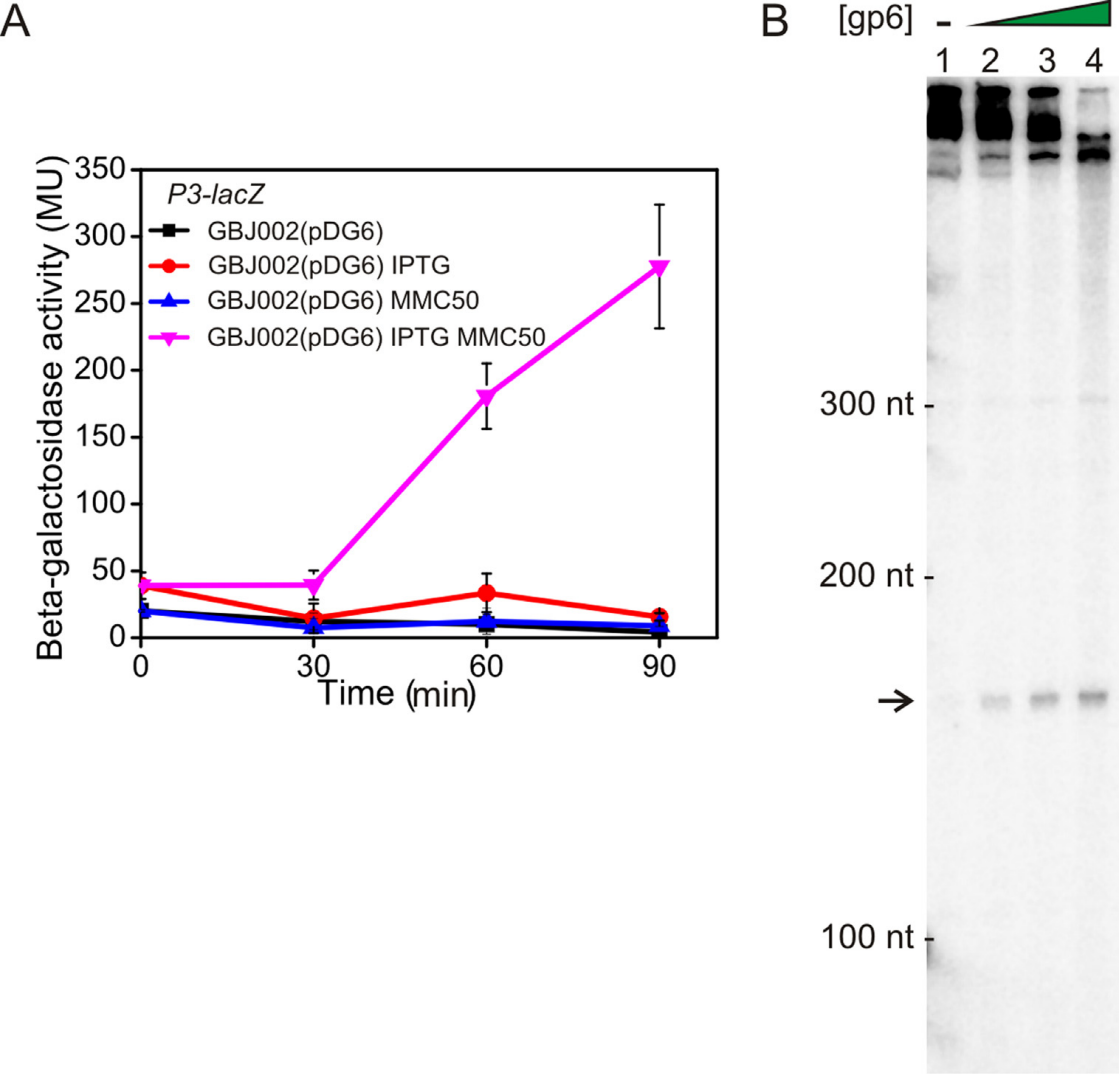
Gp6 activates transcription from the *P3* lytic promoter. (**A**) The panel shows measured β-galactosidase activities from *Bacillus thuringiensis* GBJ002 cells, which have been cured of GIL01, and carry a *P3*–*lacZ* transcriptional fusion. Cells also carried plasmid pDG6, which expresses gp6 from an IPTG-inducible promoter. To induce the SOS response, a sub-inhibitory concentration of mitomycin C (MMC50, 50 ng/ml) was added to exponentially growing cells at time 0. Expression of gp6 was induced 2 h before mitomycin C exposure by the addition of 0.1 mM IPTG. Each value is the mean ± SD of three independent measurements. (**B**) The panel shows an *in vitro* transcription assay using plasmid pSR/GIL01 *P3*, which carries the *P3* promoter. Transcription was initiated from *P3* by the addition of purified *Bacillus subtilis* RNA polymerase, carrying the SigA sigma factor, and proceeds to the strong *λoop* transcription terminator within pSR/GIL01 *P3*, producing a single 156-nt long transcript (indicated by an arrow). The concentration of gp6 in lanes 1–4 was 0, 0.3, 0.6 and 1.35 μM, respectively. All lanes contain 30 nM *B. subtilis* RNA polymerase. A representative gel is shown and experiments were performed in triplicate. Note that the large transcripts at the top of the gel result from RNA polymerase initiating transcription from other promoter sites on the pSR plasmid, which is used as a template. These produce larger transcripts than the *P3* transcript and so have more radioactivity incorporated and are more prominent. In the presence of gp6, RNA polymerase is directed to the *P3* promoter, leaving less RNA polymerase to initiate transcription from other promoter sites on the plasmid (lane 4) and this results in a decrease in the level of these larger transcripts.

Of the 8 early genes involved in GIL01 regulation and replication (Figure 1A), ORF1 and ORF6 are the only genes with predicted regulatory functions, as their products possess obvious DNA binding motifs (10,16). Since ORF1 has been linked to establishing and maintaining the lysogenic cycle (11), we examined the role of the ORF6 gene product, gp6, in the GIL01 lytic/lysogenic switch. To investigate whether gp6 regulates the *P3* promoter, we examined the expression from the *P3–lacZ* construct in *B. thuringiensis* strain GBJ002, which has been cured of GIL01, when gp6 was expressed from an IPTG-inducible promoter. Figure 3A shows that gp6 expression alone was insufficient to induce transcription from *P3*. However, when coupled with DNA damage, induced by mitomycin C exposure, gp6 expression dramatically triggered transcription from *P3* by up to 11-fold. To confirm that phage-encoded gp6 directly activates transcription at the *P3* promoter, we purified recombinant gp6 protein and performed *in vitro* transcription assays, catalyzed by the *B. subtilis* RNA polymerase holoenzyme, carrying the SigA sigma factor (18). Results in Figure 3B show that the expected 156-nt transcript was generated from *P3* in the presence, but not in the absence, of gp6, confirming that gp6 is a direct activator of transcription at *P3*. Thus, our data indicate that expression from the *P3* lytic promoter requires two signals, DNA damage, which relieves LexA binding, and expression of gp6, which is required to activate transcription initiation at *P3*.

### The gp6 activator binds to a DNA site overlapping the −35 promoter element

**Figure 4.**
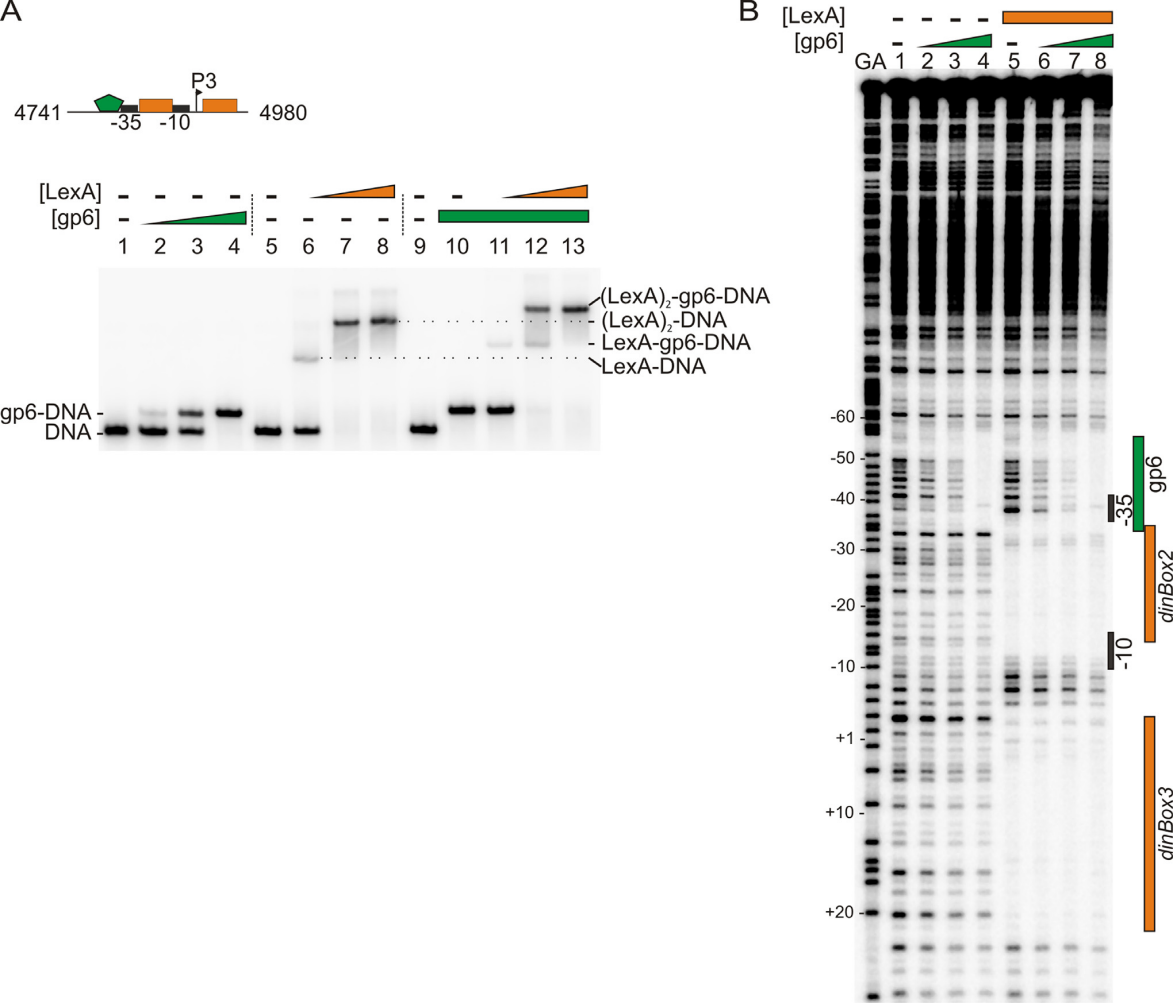
LexA and gp6 can simultaneously bind to the *P3* promoter region. (**A**) The binding of purified LexA and gp6 protein to a P^32^ end-labelled *P3* promoter fragment was assayed using EMSA. The concentration of LexA used was 0.2, 0.4 and 0.8 μM in lanes 6–8 and 11–13, respectively. The concentration of gp6 used was 0.3, 0.6, 1.35 μM in lanes 2–4 and 1.35 μM in lanes 10–13. The location of free DNA and the gp6, LexA and LexA–gp6–DNA complexes is marked. Above the EMSA is a schematic representation of the *P3* promoter fragment used for this EMSA analysis. The promoter elements are marked with black boxes, orange boxes show the position of LexA *dinBox*2 and *dinBox*3, and the green pentagon represents the gp6-binding site. The GIL01 genome coordinates of this fragment are also given. (**B**) A DNase I footprint experiment investigating the binding of gp6, in the presence or absence of LexA, to a P^32^ end-labelled *P3* promoter fragment (GIL01 genome coordinates 4801–5000). The gel was calibrated using Maxam–Gilbert G+A sequencing reactions of the labelled fragment (designated as GA), and selected positions are indicated. The concentration of gp6 used in lanes 2–4 and 6–8 was 0.3, 0.6 and 1.35 μM and the concentration of LexA used in lanes 5–8 was 0.8 μM. The location of the gp6 and LexA-binding sites and the *P3* promoter elements are shown.

**Figure 5.**
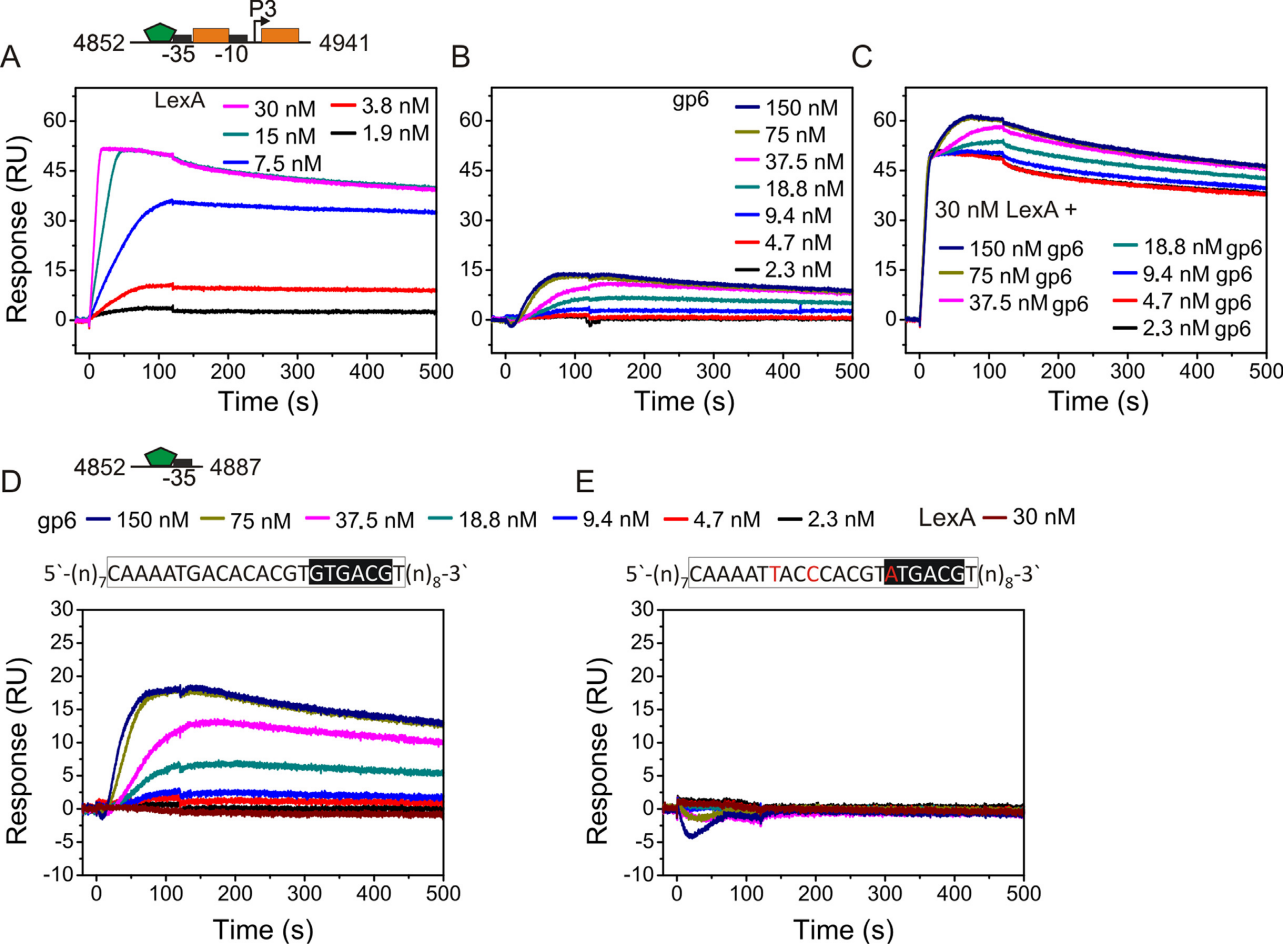
Real-time analysis of LexA and gp6 binding to the *P3* promoter. The figure shows SPR sensorgrams of (**A**) LexA, (**B**) gp6 and (**C**) LexA and gp6 binding to an immobilized *P3* promoter fragment. Sensorgrams are also shown of gp6 binding a shorter 36 bp DNA fragment, carrying the (**D**) wild-type and (**E**) a mutated gp6 target site. Proteins were injected over the immobilized DNA (∼30 RU) for 120 s at 100 μl/min. The DNA fragments used in these experiments are schematically represented above the graphs. The promoter elements are marked with black boxes, orange boxes show the position of LexA *dinBox*2 and *dinBox*3, and the green pentagon represents the gp6-binding site. The GIL01 genome coordinates of fragments are also given. In panels (D) and (E) the gp6-binding site, as determined by DNase I footprint analysis, is shown boxed, and in (E), base substitutions are shown in red. In each case, representative sensorgrams are shown and the experiments were performed in duplicate.

To investigate the interaction of gp6 with the *P3* promoter region, we examined the binding of purified gp6 and LexA proteins to a 240-bp *P3* promoter fragment, using EMSA analysis. Results in Figure 4A show that gp6 bound to the *P3* promoter DNA and that both gp6 and LexA can bind to the promoter simultaneously as super-shifted species were detected (Figure 4A: lanes 11–13). To identify the gp6-binding site at the *P3* promoter, we used DNase I footprinting with an end-labelled *P3* promoter fragment and purified gp6 and LexA protein. Results in Figure 4B show that gp6 binds to a sequence overlapping with the potential −35 element (lanes, 2–4), which is a typical location for a class II activator to bind and interact directly with RNA polymerase (30). Furthermore, when LexA was present (lanes, 5–8) it protected both *dinBox*2 and *dinBox*3 and did not appear to interfere with gp6 association. It is of note that the LexA-induced hypersensitive bands observed between *dinBox*2 and *dinBox*3 are altered by the inclusion of gp6, suggesting that gp6 might subtly alter LexA binding to the *P3* promoter. In addition, we used SPR analysis to examine the concurrent binding of gp6 and LexA to a DNA probe, carrying the *P3* −35 element and the downstream *dinBox*2 and *dinBox*3 boxes (Figure 5A–C). Data in Figure 5 confirm that when saturating concentrations of both gp6 and LexA were present, the two proteins simultaneously bound *P3* without interference, as traces for concurrent binding are the sum of the values when each protein was present individually.

Inspection of the region protected by gp6 in our footprint analysis (Figure 4B) identified a palindromic sequence (GaCACAC│GTGTGaC), centered at position −43.5 upstream of the *P3* transcription start site (+1) (Figure 1A). Therefore, to identify important bases in the gp6-binding site, we introduced three substitutions into this region and monitored their effect on gp6 binding, using SPR analysis (Supplementary Figure S4; Figure 5D and E). Our results show that single point mutations decreased the stability of gp6 binding and that combining all three substitutions completely abolished binding. Thus, we have identified crucial sequences for the interaction of gp6 with the *P3* promoter region.

### The gp6 activator protein is a truncated homologue of the LexA repressor

**Figure 6.**
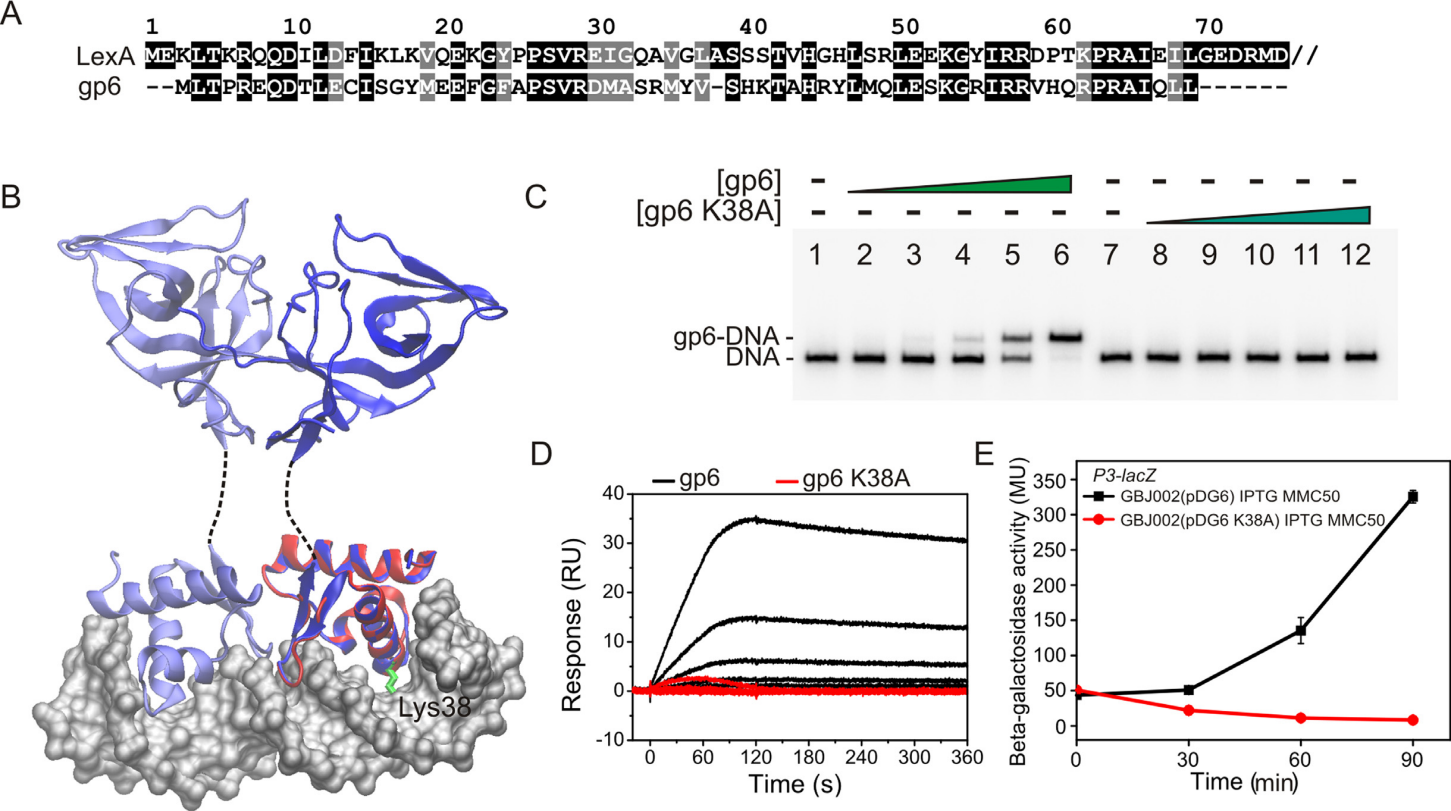
The GIL01 bacteriophage gp6 protein is homologous to the DNA-binding domain of the *Bacillus thuringiensis* LexA repressor. (**A**) The panel shows an amino-acid sequence alignment of the *B. thuringiensis* subsp. *israelensis* LexA sequence (Uniprot ID: A0A160LCW5) with the GIL01 gp6 protein sequence (Uniprot ID: Q5ILC6). Identical residues are shaded black and similar residues are in grey. Only the first 75 amino acids of the *B. thuringiensis* LexA repressor are shown. (**B**) The panel shows a three-dimensional structural model of gp6 (red) superimposed onto the crystal structure of the *Escherichia coli* LexA repressor (blue) in complex with its DNA site (PDB ID: 3jso) (25). Dashed lines between the *E. coli* LexA N- and C-terminal domains represent the flexible linker region that was not resolved in the crystal structure. For gp6, Lys38 is shown in green, protruding deep into the DNA major groove. (**C**) EMSA analysis investigating the binding of wild-type gp6 and the gp6 K38A mutant with a *P3* promoter region fragment (see Figure 4). The concentration of proteins used was 0, 0.15, 0.3, 0.6, 1.2 and 2.4 μM in lanes 1–6 and in lanes 7–12 for the gp6 and gp6 K38A, respectively. The location of free DNA and the gp6–DNA complex is marked. (**D**) The panel shows the SPR sensorgram of an experiment investigating the interaction of wild-type gp6 and the gp6 K38A protein with a 36 bp DNA fragment carrying the gp6 target site (see Figure 5D). The protein concentrations tested were 2.34, 4.69, 9.4, 18.8, 37.5, 75 or 150 nM for both proteins. Proteins were injected over the immobilized DNA (∼100 RU) for 120 s at 100 μl/min and dissociation was followed for 240 s. Representative sensorgrams are shown and the experiments were performed in duplicate. (**E**) β-Galactosidase activities from *B. thuringiensis* GBJ002 carrying a *P3*–*lacZ* transcriptional fusion and either plasmid pDG6 or pDG6 K38A, which express gp6 or the gp6 K38A derivative, respectively, from an IPTG-inducible promoter. To induce the SOS response, a sub-inhibitory concentration of mitomycin C (MMC50, 50 ng/ml) was added to exponentially growing cells at time 0. Expression of gp6 was induced 2 h before mitomycin C exposure by the addition of 0.1 mM IPTG. Each value is the mean ± SD of two independent measurements.

Bioinformatic analysis of gp6 indicated that it is a truncated LexA homologue, primarily consisting of the LexA DNA-binding domain, and alignment of gp6 with the corresponding region of *B. thuringiensis* LexA revealed that the two proteins possess 44% sequence identity in this region (Figure 6A) (31). Therefore, to gain more insight into the structure of gp6, we generated a homology model of GIL01 gp6, using the crystal structure of *Thermotoga maritima* LexA repressor (PDB ID: 3k2z). This model was then superimposed onto the structure of *E. coli* LexA bound to DNA (PDB ID: 3jso) (25), with the backbone trace of gp6 overlaid with LexA DNA-binding domain (Figure 6B). This gp6 homology model suggests that similar to LexA, gp6 is composed of a helix-turn-helix (HTH) motif containing three α-helices followed by a 14-residue C-terminus extension that folds into two-strand β-sheet to form the characteristic LexA winged HTH motif. It is of note that the side chain of lysine 38 (K38), within the gp6 HTH recognition helix, would be potentially positioned to protrude into the DNA major groove and make contacts with the target DNA sequence (Figure 6B). Therefore, to investigate this and gain support for our gp6 structural model we performed site-directed mutagenesis on gp6, changing the lysine at position 38 to alanine to generate the gp6 K38A mutant protein. The overexpression and purification of soluble gp6 K38A protein was similar to that experienced for wild-type protein; however, EMSA and SPR analysis clearly indicated that gp6 K38A was unable to interact with its binding site at the *P3* promoter (Figure 6C and D). In support of this, the expression of gp6 K38A in *B. thuringiensis* GBJ002 cells failed to activate transcription from *P3*, even when coupled with DNA damage (Figure 6E). Thus, our data strongly suggest that like LexA, gp6 is a member of the ‘winged helix’ family of DNA binding proteins and that residue K38 is important for its DNA binding site recognition.

## DISCUSSION

On infection of *B. thuringiensis*, phage GIL01 can establish a lysogenic state, which is stably maintained until its host experiences severe DNA damage (10,11). This process is controlled by the host’s SOS regulator, LexA, which represses the *P1* promoter that is responsible for driving the expression of the GIL01 early genes. To gain more insight into the biology of GIL01, we investigated the lytic/lysogenic switch in more detail by examining the regulation of the *P3* lytic promoter, which controls the expression of GIL01 capsid and host lysis genes. We show that like *P1*, the *P3* promoter is switched on by DNA damage, but that expression from *P3* is delayed in comparison. We show that this regulation is achieved by the coordinated interplay between host-encoded LexA and two small phage proteins, gp7 and gp6, at the *P3* promoter region and propose that this complex regulation is required to ensure timely expression of the GIL01 late genes.

Previously, we demonstrated that at the *P1* promoter, LexA formed a complex with GIL01-encoded gp7 to enable LexA to bind to the poorly conserved, low-affinity LexA box *dinBox*1b and, thus, repress transcription (12). Our SPR analysis and DNase I footprint experiments at the *P3* promoter indicate that gp7 can also interact with LexA at high-affinity sites (i.e.*dinBox*2 and *dinBox*3) (Figure 2 and Supplementary Figure S2). Experiments in which the spacing between *dinBox*2 and *dinBox*3 was altered, moving these sites around the DNA helix (Supplementary Figure S3) had no effect on the binding of the LexA–gp7 complex and indicated that complex formation is independent of operator spacing. Since gp7 has been shown to also interact with the well conserved *lexA* and *recA* promoters in *B. thuringiensis*, this suggests that such a LexA–gp7 complex likely affects all LexA-regulated promoters within the cell (12). Interestingly, gp7 is found in the genomes of many Tectiviruses (Supplementary Figure S5), some of which are carried by important human pathogens (e.g.*B. cereus, B. anthracis* and *Streptococcus pneumoniae*). Thus, it is possible that gp7 modulation of LexA binding is a common mechanism used by this family of phage to modify the SOS response of their host.

Experiments with a *P3*–*lacZ* fusion in the GIL01-cured host (Figures 1 and 3) demonstrated that, in addition to DNA damage, the phage-borne factor, gp6, was required to directly activate transcription from the *P3* promoter. Analysis of gp6 and LexA binding (Figures 4 and 5) indicated that both proteins could bind to *P3* simultaneously, without interference, and that gp6 binds to an upstream sequence overlapping the proposed *P3* −35 element sequence, centered at position −43.5. The positioning of this motif suggests that gp6 activates transcription by interacting directly with RNA polymerase to aid recruitment of polymerase to the promoter DNA, as is observed for other transcription factors (30). Intriguingly, gp6 is homologous to the N-terminal DNA binding domain of LexA (Figure 6). Thus, at the *P3* promoter two LexA family members, with opposing functions, control transcription. Like gp7, gp6 homologues are found in a number of Tectiviruses genomes (Supplementary Figure S6), suggesting that the gp6 coding sequence was originally acquired from a bacterial host and evolved to recognize a distinct nucleotide sequence at *P3* and activate transcription. It is of note that although LexA represses transcription at the majority of promoters, examples of LexA-mediated activation have been observed (32–34). The widespread occurrence of gp6 in various *Bacillus* species (Supplementary Figure S6) also suggests that the activation of late gene expression by gp6 homologues may be a common mechanism of regulation in temperate Tectiviruses. In accordance with this, the gp6 palindromic binding sequence identified at *P3* (GaCACAn│nTGTGaC) is conserved in many Tectiviruses (35) (Supplementary Figure S7) as is the lysine at position 38 (K38) in gp6, which we demonstrate is important for gp6 binding to *P3* (Figure 6 and Supplementary Figure S6).

**Figure 7.**
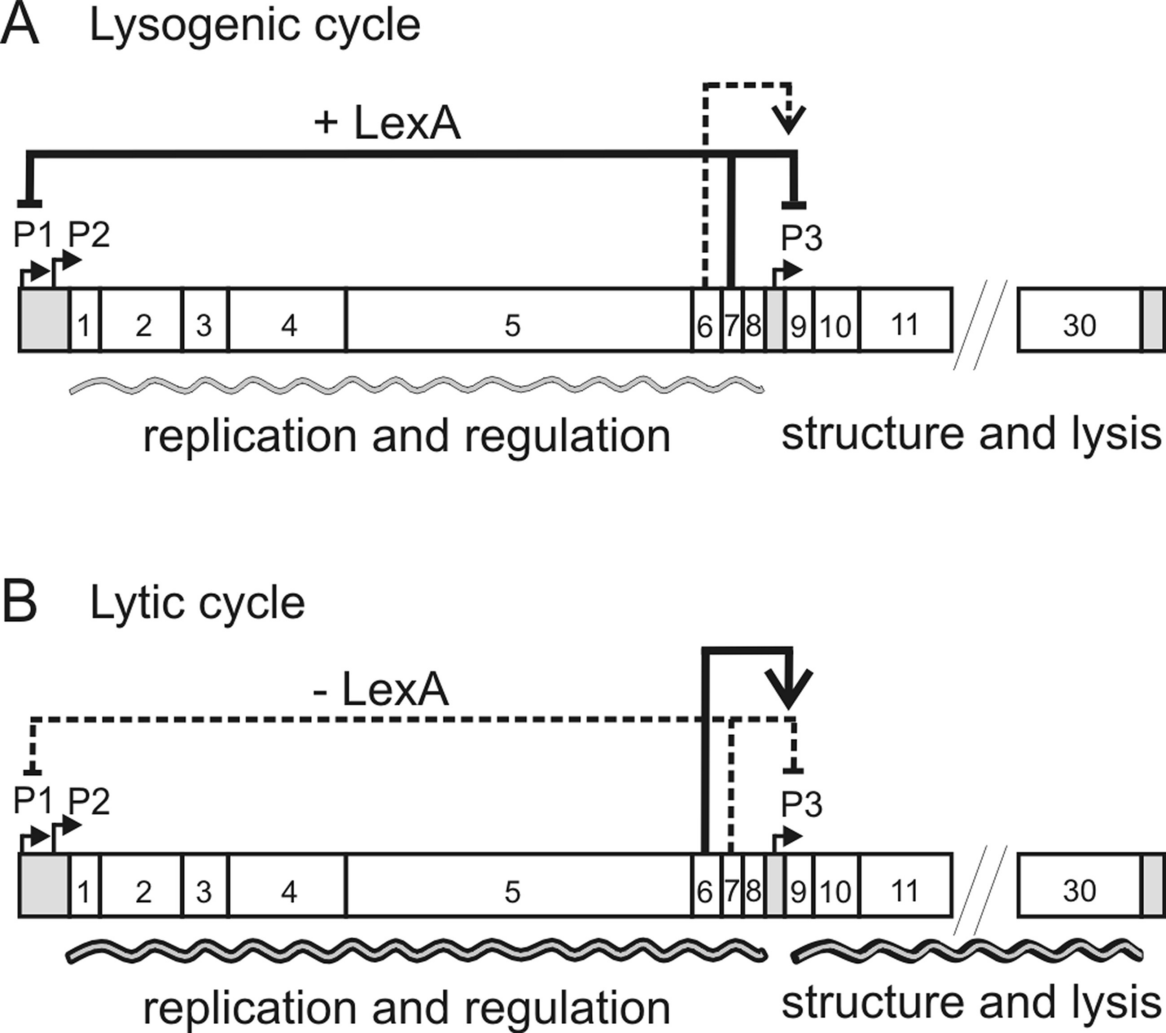
Regulation of the lytic/lysogenic switch in the *Bacillus thuringiensis* temperate phage GIL01. The figure shows the genetic map of GIL01, highlighting key genes and regulatory sites. (**A**) Maintenance of the GIL01 lysogenic state. Host LexA protein, in conjunction with the product of ORF7, gp7, represses the expression of phage functions directed from the *P1, P2* and *P3* promoters to maintain lysogeny. (**B**) The lytic cycle. Upon persistent DNA damage, LexA undergoes auto-cleavage and its cellular concentration drops below a threshold level, which results in derepression of *P1* and *P2*, and high-level expression of the replication and regulatory genes to initiate the lytic cycle. Substantial intracellular accumulation of gp6 protein activates transcription from *P3*, (located between ORF8 and ORF9), resulting in the expression of the downstream phage structural and lysis genes and eventual host cell lysis and death.

Based on our data, we propose a regulatory model to explain how GIL01 is able to switch from a dormant lysogen into the lytic cycle (Figure 7). We propose that in unstressed cells, low-level expression from the *P1* promoter, and possibly *P2*, leads to the expression of gp7 and other early gene products. However, the association of gp7 with LexA further represses *P1* and keeps levels of gp6 below the threshold required for *P3* induction, maintaining lysogeny (Figure 7A). After significant and sustained DNA damage (Figure 7B), intracellular levels of LexA drop considerably, leading to the dissociation of LexA from the *P1* and *P3* promoter regions. The *P1* promoter is now fully active and high-level expression and accumulation of gp6 ensue. Once gp6 levels are sufficient, gp6 can directly activate transcription at *P3*, resulting in the expression of GIL01 late genes and the eventual lysis and death of the host cell.

The delayed expression of the GIL01 late genes from *P3* is reminiscent of the expression profile observed for the colicin E8 (*cea8*) and colicin K (*cka*) genes in *E. coli* (13,36,37). For both the GIL01 late genes and colicin operons, product expression is suicidal for the host bacterium and so, the promoters controlling their expression are tightly repressed. In the case of colicins, expression is delayed due to co-regulation by LexA and an additional host repressor (e.g. AsnC or IscR), which ensures that their production only occurs after prolonged DNA damage and nutrient starvation (13,36,37). For the GIL01 *P3* promoter an alternative mechanism has evolved, which ensures that late gene expression is dependent both on DNA damage, via LexA, and expression of an early phage gene, gp6. In addition to this, GIL01 deploys gp7 to directly modulate the host’s response to DNA damage, to affect both phage and host LexA-promoters alike. Recently, it has been shown that the modulation of LexA can affect the response of bacteria to certain antibiotics (38). Thus, the widespread occurrence of virally encoded gp7 homologues in the genomes of various human pathogens could mean that lysogeny by Tectiviruses has important implications for antibiotic resistance in these important bacterial species.

## Supplementary Material

Supplementary Data

## References

[1] Feiner R., Argov T., Rabinovich L., Sigal N., Borovok I., Herskovits A.A. A new perspective on lysogeny: prophages as active regulatory switches of bacteria. Nat. Rev. Microbiol. 2015; 13:641–650. 26373372 10.1038/nrmicro3527

[2] Benson S.D., Bamford J.K., Bamford D.H., Burnett R.M. Viral evolution revealed by bacteriophage PRD1 and human adenovirus coat protein structures. Cell. 1999; 98:825–833. 10499799 10.1016/s0092-8674(00)81516-0

[3] Krupovic M., Koonin E. V. Polintons: a hotbed of eukaryotic virus, transposon and plasmid evolution. Nat. Rev. Microbiol. 2014; 13:105–115. 25534808 10.1038/nrmicro3389 PMC5898198

[4] Abrescia N.G.A., Cockburn J.J.B., Grimes J.M., Sutton G.C., Diprose J.M., Butcher S.J., Fuller S.D., San Martín C., Burnett R.M., Stuart D.I. Insights into assembly from structural analysis of bacteriophage PRD1. Nature. 2004; 432:68–74. 15525981 10.1038/nature03056

[5] Saren A.-M., Ravantti J.J., Benson S.D., Burnett R.M., Paulin L., Bamford D.H., Bamford J.K.H. A snapshot of viral evolution from genome analysis of the tectiviridae family. J. Mol. Biol. 2005; 350:427–440. 15946683 10.1016/j.jmb.2005.04.059

[6] Yutin N., Bäckström D., Ettema T.J.G., Krupovic M., Koonin E. V. Vast diversity of prokaryotic virus genomes encoding double jelly-roll major capsid proteins uncovered by genomic and metagenomic sequence analysis. Virol. J. 2018; 15:67. 29636073 10.1186/s12985-018-0974-y PMC5894146

[7] Jalasvuori M., Koskinen K. Extending the hosts of Tectiviridae into four additional genera of Gram-positive bacteria and more diverse *Bacillus* species. Virology. 2018; 518:136–142. 29481984 10.1016/j.virol.2018.02.014

[8] Gill J.J., Wang B., Sestak E., Young R., Chu K.-H. Characterization of a novel Tectivirus phage Toil and its potential as an agent for biolipid extraction. Sci. Rep. 2018; 8:1062. 29348539 10.1038/s41598-018-19455-2 PMC5773508

[9] Gillis A., Mahillon J. Phages preying on *Bacillus anthracis, Bacillus cereus*, and *Bacillus thuringiensis*: past, present and future. Viruses. 2014; 6:2623–2672. 25010767 10.3390/v6072623 PMC4113786

[10] Verheust C., Jensen G., Mahillon J. pGIL01, a linear tectiviral plasmid prophage originating from *Bacillus thuringiensis* serovar *israelensis*. Microbiology. 2003; 149:2083–2092. 12904548 10.1099/mic.0.26307-0

[11] Fornelos N., Bamford J.K., Mahillon J. Phage-borne factors and host LexA regulate the lytic switch in phage GIL01. J. Bacteriol. 2011; 193:6008–6019. 21890699 10.1128/JB.05618-11 PMC3194922

[12] Fornelos N., Butala M., Hodnik V., Anderluh G., Bamford J.K., Salas M. Bacteriophage GIL01 gp7 interacts with host LexA repressor to enhance DNA binding and inhibit RecA-mediated auto-cleavage. Nucleic Acids Res. 2015; 43:7315–7329. 26138485 10.1093/nar/gkv634 PMC4551915

[13] Fornelos N., Browning D.F., Butala M. The use and abuse of LexA by mobile genetic elements. Trends Microbiol. 2016; 24:391–401. 26970840 10.1016/j.tim.2016.02.009

[14] Butala M., Žgur-Bertok D., Busby S.J. The bacterial LexA transcriptional repressor. Cell. Mol. Life Sci. 2009; 66:82–93. 18726173 10.1007/s00018-008-8378-6 PMC11131485

[15] Erill I., Campoy S., Barbe J. Aeons of distress: an evolutionary perspective on the bacterial SOS response. FEMS Microbiol. Rev. 2007; 31:637–656. 17883408 10.1111/j.1574-6976.2007.00082.x

[16] Berjón-Otero M., Lechuga A., Mehla J., Uetz P., Salas M., Redrejo-Rodríguez M. Bam35 tectivirus intraviral interaction map unveils new function and localization of phage ORFan proteins. J. Virol. 2017; 91:e00870-17. 10.1128/JVI.00870-17 PMC5599771 28747494

[17] Jensen G.B., Andrup L., Wilcks A., Smidt L., Poulsen O.M. The aggregation-mediated conjugation system of *Bacillus thuringiensis* subsp. *israelensis*: host range and kinetics of transfer. Curr. Microbiol. 1996; 33:228–236. 8824168 10.1007/s002849900105

[18] Rabatinová A., Šanderová H., Jirát Matějčková J., Korelusová J., Sojka L., Barvík I., Papoušková V., Sklenár V., Žídek L., Krásný L. The δ subunit of RNA polymerase is required for rapid changes in gene expression and competitive fitness of the cell. J. Bacteriol. 2013; 195:2603–2611. 23543716 10.1128/JB.00188-13 PMC3676059

[19] Lereclus D., Agaisse H., Gominet M., Salamitou S., Sanchis V. Identification of a *Bacillus thuringiensis* gene that positively regulates transcription of the phosphatidylinositol-specific phospholipase C gene at the onset of the stationary phase. J. Bacteriol. 1996; 178:2749–2756. 8631661 10.1128/jb.178.10.2749-2756.1996 PMC178008

[20] Rossiter A.E., Godfrey R.E., Connolly J.A., Busby S.J.W., Henderson I.R., Browning D.F. Expression of different bacterial cytotoxins is controlled by two global transcription factors, CRP and Fis, that co-operate in a shared-recruitment mechanism. Biochem. J. 2015; 466:323–335. 25484033 10.1042/BJ20141315

[21] Browning D.F., Lee D.J., Wolfe A.J., Cole J.A., Busby S.J.W. The *Escherichia coli* K-12 NarL and NarP proteins insulate the *nrf* promoter from the effects of integration host factor. J. Bacteriol. 2006; 188:7449–7456. 16936015 10.1128/JB.00975-06 PMC1636288

[22] Godfrey R.E., Lee D.J., Busby S.J.W., Browning D.F. Regulation of *nrf* operon expression in pathogenic enteric bacteria: sequence divergence reveals new regulatory complexity. Mol. Microbiol. 2017; 104:580–594. 28211111 10.1111/mmi.13647 PMC5434802

[23] Sievers F., Wilm A., Dineen D., Gibson T.J., Karplus K., Li W., Lopez R., McWilliam H., Remmert M., Söding J. Fast, scalable generation of high-quality protein multiple sequence alignments using Clustal Omega. Mol. Syst. Biol. 2011; 7:539. 21988835 10.1038/msb.2011.75 PMC3261699

[24] Biasini M., Bienert S., Waterhouse A., Arnold K., Studer G., Schmidt T., Kiefer F., Cassarino T.G., Bertoni M., Bordoli L. SWISS-MODEL: modelling protein tertiary and quaternary structure using evolutionary information. Nucleic Acids Res. 2014; 42:W252–W258. 24782522 10.1093/nar/gku340 PMC4086089

[25] Zhang A.P., Pigli Y.Z., Rice P.A. Structure of the LexA-DNA complex and implications for SOS box measurement. Nature. 2010; 466:883–886. 20703307 10.1038/nature09200 PMC2921665

[26] Humphrey W., Dalke A., Schulten K. VMD: visual molecular dynamics. J. Mol. Graph. 1996; 14:33–38. 8744570 10.1016/0263-7855(96)00018-5

[27] Courcelle J., Khodursky A., Peter B., Brown P.O., Hanawalt P.C. Comparative gene expression profiles following UV exposure in wild-type and SOS-deficient *Escherichia coli*. Genetics. 2001; 158:41–64. 11333217 10.1093/genetics/158.1.41 PMC1461638

[28] Craig M.L., Suh W.C., Record M.T. Jr. HO. and DNase I probing of E sigma 70 RNA polymerase–lambda PR promoter open complexes: Mg^2+^ binding and its structural consequences at the transcription start site. Biochemistry. 1995; 34:15624–15632. 7495790 10.1021/bi00048a004

[29] Kuhner F., Costa L.T., Bisch P.M., Thalhammer S., Heckl W.M., Gaub H.E. LexA-DNA bond strength by single molecule force spectroscopy. Biophys. J. 2004; 87:2683–2690. 15454462 10.1529/biophysj.104.048868 PMC1304687

[30] Browning D.F., Busby S.J.W. Local and global regulation of transcription initiation in bacteria. Nat. Rev. Microbiol. 2016; 14:638–650. 27498839 10.1038/nrmicro.2016.103

[31] Verheust C., Fornelos N., Mahillon J. GIL16, a new Gram-positive Tectiviral Phage related to the *Bacillus thuringiensis* GIL01 and the *Bacillus cereus* pBClin15 elements. J. Bacteriol. 2005; 187:1966–1973. 15743944 10.1128/JB.187.6.1966-1973.2005 PMC1064052

[32] Tapias A., Fernandez S., Alonso J.C., Barbe J. *Rhodobacter sphaeroides* LexA has dual activity: optimising and repressing *recA* gene transcription. Nucleic Acids Res. 2002; 30:1539–1546. 11917014 10.1093/nar/30.7.1539 PMC101838

[33] Gutekunst K., Phunpruch S., Schwarz C., Schuchardt S., Schulz-Friedrich R., Appel J. LexA regulates the bidirectional hydrogenase in the cyanobacterium *Synechocystis* sp. PCC 6803 as a transcription activator. Mol. Microbiol. 2005; 58:810–823. 16238629 10.1111/j.1365-2958.2005.04867.x

[34] Kimsey H.H., Waldor M.K. *Vibrio cholerae* LexA coordinates CTX prophage gene expression. J. Bacteriol. 2009; 191:6788–6795. 19666711 10.1128/JB.00682-09 PMC2772474

[35] Jalasvuori M., Palmu S., Gillis A., Kokko H., Mahillon J., Bamford J.K.H., Fornelos N. Identification of five novel tectiviruses in *Bacillus* strains: analysis of a highly variable region generating genetic diversity. Res. Microbiol. 2013; 164:118–126. 23103336 10.1016/j.resmic.2012.10.011

[36] Kamenšek S., Browning D.F., Podlesek Z., Busby S.J.W., Žgur-Bertok D., Butala M. Silencing of DNase colicin E8 gene expression by a complex nucleoprotein assembly ensures timely colicin induction. PLoS Genet. 2015; 11:e1005354. 26114960 10.1371/journal.pgen.1005354 PMC4482635

[37] Butala M., Sonjak S., Kamenšek S., Hodošček M., Browning D.F., Žgur-Bertok D., Busby S.J.W. Double locking of an *Escherichia coli* promoter by two repressors prevents premature colicin expression and cell lysis. Mol. Microbiol. 2012; 86:129–139. 22812562 10.1111/j.1365-2958.2012.08179.x

[38] Mo C.Y., Culyba M.J., Selwood T., Kubiak J.M., Hostetler Z.M., Jurewicz A.J., Keller P.M., Pope A.J., Quinn A., Schneck J. Inhibitors of LexA autoproteolysis and the bacterial SOS response discovered by an academic-industry partnership. ACS Infect. Dis. 2018; 4:349–359. 29275629 10.1021/acsinfecdis.7b00122 PMC5893282

