# Supplementary material for: Lytic gene expression in the temperate bacteriophage GIL01 is activated by a phage-encoded LexA homologue
Source: Nucleic Acids Res. 2018 Jul 24;46(18):9432–43. doi: 10.1093/nar/gky646 (PMC6182141; doi:10.1093/nar/gky646)
Supplement: Supplementary Data [file gky646_supplemental_files.docx]

**Supplementary Data**

**Lytic gene expression in the temperate bacteriophage GIL01 is activated by a phage-encoded LexA homologue.**

Nadine Fornelos*, Douglas F. Browning*, Anja Pavlin, Zdravko Podlesek, Vesna Hodnik, Margarita Salas, Matej Butala*

*To whom correspondence should be addressed:

MB: Email: matej.butala@bf.uni-lj.si Tel: +386 (1)320-33-97.

*Correspondence may also be addressed to:

NF: Email: nadinefm@broadinstitute.org Tel: +1 617-714 8205.

DFB: Email: D.F.Browning@bham.ac.uk Tel: +44 (0)121-414-5435.

Supplementary Table S1. Bacterial strains and plasmids used in this study.

| **Bacterial strain/plasmid** | **Description** | **Source** |  |
| --- | --- | --- | --- |
| *B. thuringiensis* serovar *israelensis* GBJ002 | Natural host of phage GIL01, a derivative of strain 4Q2 cured of all plasmids and GIL01. | (1, 2) |  |
| *B. thuringiensis* serovar *israelensis* GBJ338 | GIL01 lysogen of strain GBJ002. | (1) |  |
| *E. coli* M15 pREP4 | Expression host, carrying the pREP4 plasmid, which is used in combination with IPTG-inducible pQE vectors for protein over-expression; F^-^, Φ80*Δlac*M15, *thi*, *lac^-^*, *mtl^-^*, *recA*^+^ , Km^R^ | Qiagen |  |
| *E. coli* BL21(DE3) pLysE | Expression host, harboring the lambda DE3 lysogen and the pLysE plasmid, which constitutively expresses T7 lysozyme. Used in combination with pET vectors for protein over-expression;  *E. coli* str. B F^–^ *ompT* *gal* *dcm* *lon* *hsdS_B_*(*r_B_*^–^*m_B_*^–^) λ(DE3 [*lacI* *lacUV5*-*T7p07* *ind1* *sam7* *nin5*]) [*malB*^+^]_K-12_(λ^S^) pLysS[*T7p20* *ori*_p15A_](Cm^R^) | Thermo Fischer Scientific |  |
| *E. coli* JW3350 | *rrnB3* Δ*lacZ4787 hsdR514* Δ(*araBAD*)*567* Δ(*rhaBAD)568 rph-1 dam* | (3) |  |
| pHT304-18Z | A low-copy number shuttle vector, carrying a promotor less *lacZ* gene. Ap^R^ in *E. coli* and Ery^R^ in *B. thuringiensis*. | (4) |  |
| pDin1 | pHT304-18Z, carrying a *P1*-*lacZ* promoter fusion, Ery^R^ | (5) |  |
| pDin3 | pHT304-18Z, carrying a *P3*-*lacZ* promoter fusion, Ery^R^ | (1) |  |
| pET8c | *E. coli* T7 expression plasmid, which carries an N-terminal hexahistidine tag (His_6_) and thrombin cleavage site. | Novagen |  |
| pgp6 | A pET8c derivative for the over-expression of His_6_-gp6 in *E. coli* BL21(DE3), Ap^R^ | This study |  |
| pgp6 K38A | A pET8c derivative for the over-expression of His_6_-gp6 K38A in *E. coli* BL21(DE3), Ap^R^ | This study |  |
| pDG148 | Shuttle vector for IPTG-inducible gene expression in *Bacillus subtilis,* Km^R^, Ap^R^ | (6) |  |
| pDG6 | A pDG148 derivative, carrying GIL01 ORF6, for IPTG-induced expression of His_6_-gp6 in *B. thuringiensis*, Km^R^, Ap^R^ | This study |  |
| pDG6 K38A | A pDG148 derivative, carrying GIL01 ORF6, for IPTG-induced expression of His_6_-gp6 K38A in *B. thuringiensis*, Km^R^, Ap^R^ | This study |  |
| pDG7 | A pDG148 derivative, carrying ORF7, for IPTG-induced expression of His_6_-gp7 in *B. thuringiensis*, Km^R^, Ap^R^ | (1) |  |
| pQELexA | An IPTG-inducible *lexA* expression plasmid, derived from pQE-30, for the expression of His_6_-LexA in *E. coli* M15 pREP4, Km^R^, Ap^R^ | (5) |  |
| pQE7 | An IPTG-inducible gp7 expression plasmid, derived from pQE-30, for the expression of His_6_-gp7 in *E. coli* M15 pREP4, Km^R^, Ap^R^ | (1) |  |
| pSR | A pBR322 derivative containing the λ*oop* transcription terminator, Ap^R^ | (7) |  |
| pSR/ GIL01  *P3* | A pSR derivative carrying the *P3* promoter region upstream of the λ*oop* transcription terminator, Ap^R^ | This study |  |

Supplementary Table S2. Primers used in this study.

| **Primers** | Sequence* |  |
| --- | --- | --- |
| gp6_u | CGCGGATCCTTGACGCCAAGGGAACAGGATACG |  |
| gp6_d | CGCACGCGTTTATAACAACTGGA |  |
| gp6BT_u | TCTAGATGTTGACGCCAAGGGAACAG |  |
| gp6BT_d | GCATGCTTATAACAACTGGATGGCTC |  |
| K38A_u | GCGATGTGCTGTCGCATGACTTACGTACATTCGACTCGC |  |
| K38A_d | GCGAGTCGAATGTACGTAAGTCATGCGACAGCACATCGC |  |
| GILp3(UP) | GGGGGAATTCAGCATTAAGGAGACAAGCAAAACCG |  |
| GILp3(Down) | GGGGAAGCTTTAGCTATGATTAGTTCCGTTAAAGC |  |
| B23_wt_u | GTTACTACTCGAGCGCGTTATGCGGAACACTCGTTCGTATTATAGTATACATATAGCGAACAAACATTCGATAAGGAG |  |
| B23_wt_d | CTCCTTATCGAATGTTTGTTCGCTATATGTATACTATAATACGAACGAGTGTTCCGCATAACG |  |
| B23_m1_u | GTTACTACTCGAGCGCGTTATGCG**T**A**G**CACTCGT**G**CGTATTATAGTATACATATAGCGAACAAACATTCGATAAGGAG |  |
| B23_m1_d | CTCCTTATCGAATGTTTGTTCGCTATATGTATACTATAATACGCACGAGTGCTACGCATAACG |  |
| B23_m2_u | GTTACTACTCGAGCGCGTTATGCGGAACACTCGTTCGTATTATAGTATACATATAGC**T**A**G**CAAACAT**G**CGATAAGGAG |  |
| B23_m2_d | CTCCTTATCGCATGTTTGCTAGCTATATGTATACTATAATACGAACGAGTGTTCCGCATAACG |  |
| B23_ns_u | GTTACTACTCGAGCGCGTTATGCG**T**A**G**CACTCGT**G**CGTATTATAGTATACATATAGC**T**A**G**CAAACAT**G**CGATAAGGAG |  |
| B23_ns_d | CTCCTTATCGCATGTTTGCTAGCTATATGTATACTATAATACGCACGAGTGCTACGCATAACG |  |
| B23_Lwt_u | GTTACTACTCGAGCGATTTGTCCAAAATGACACACGTGTGACGTTATGCGGAACACTCGTTCGTATTATAGTATACATATAGCGAACAAACATTCGATAAGGAG |  |
| B23_Lwt_d | CTCCTTATCGAATGTTTGTTCGCTATATGTATACTATAATACGAACGAGTGTTCCGCATAACGTCACACGTGTGTCATTTTGGACAAAT |  |
| B23_5ex_u | GTTACTACTCGAGCGCGTTATGCGGAACACTCGTTCGTATTATAGTatataATACATATAGCGAACAAACATTCGATAAGGAG |  |
| B23_5ex_d | CTCCTTATCGAATGTTTGTTCGCTATATGTATtatatACTATAATACGAACGAGTGTTCCGCATAACG |  |
| B23_10ex_u | GTTACTACTCGAGCGCGTTATGCGGAACACTCGTTCGTATTATAGTatatctatatATACATATAGCGAACAAACATTCGATAAGGAG |  |
| B23_10ex_d | CTCCTTATCGAATGTTTGTTCGCTATATGTATtatagatatACTATAATACGAACGAGTGTTCCGCATAACG |  |
| B23_Swt_u | GTTACTACTCGAGCGATTTGTCCAAAATGACACACGTGTGACGTTATGCGG |  |
| B23_Swt_d | CCGCATAACGTCACACGTGTGTCATTTTGGACAAAT |  |
| B23_Sm1_u | GTTACTACTCGAGCGATTTGTCCAAAAT**T**ACACACGTGTGACGTTATGCGG |  |
| B23_Sm1_d | CCGCATAACGTCACACGTGTGT**A**ATTTTGGACAAAT |  |
| B23_Sm2_u | GTTACTACTCGAGCGATTTGTCCAAAATGAC**C**CACGTGTGACGTTATGCGG |  |
| B23_Sm2_d | CCGCATAACGTCACACGTG**G**GTCATTTTGGACAAAT |  |
| B23_Sm3_u | GTTACTACTCGAGCGATTTGTCCAAAATGACACACGT**A**TGACGTTATGCGG |  |
| B23_Sm3_d | CCGCATAACGTCA**T**ACGTGTGTCATTTTGGACAAAT |  |
| B23_Smns_u | GTTACTACTCGAGCGATTTGTCCAAAAT**T**AC**C**CACGT**A**TGACGTTATGCGG |  |
| B23_Smns_d | CCGCATAACGTCA**T**ACGTG**G**GT**A**ATTTTGGACAAAT |  |
| EMSA7 (4801) | TGTTGAACAAGGAATAGTAG |  |
| EMSA8 (4918) | CTATATGTATACTATAATACGAAC |  |
| EMSA9 (4900) | TATTATAGTATACATATAGCGAAC |  |
| EMSA10 (5000) | AAGCATTTCCACTTTATTG |  |
| S1 | CGCTCGAGTAGTAAC-TEG-Bio |  |

* All primer sequences are shown in the 5’‑3’ direction. Restriction sites are underlined and sequences that anneal to the immobilized SPR S1 primer are double underlined. SOS boxes are shown by dashed lines and the gp6-binding sequences are boxed. Substitutions are presented in boldface and inserted nucleotides, between SOS boxes, are shown as lower case. Bio denotes a biotin label and TEG is triethylene glycol.


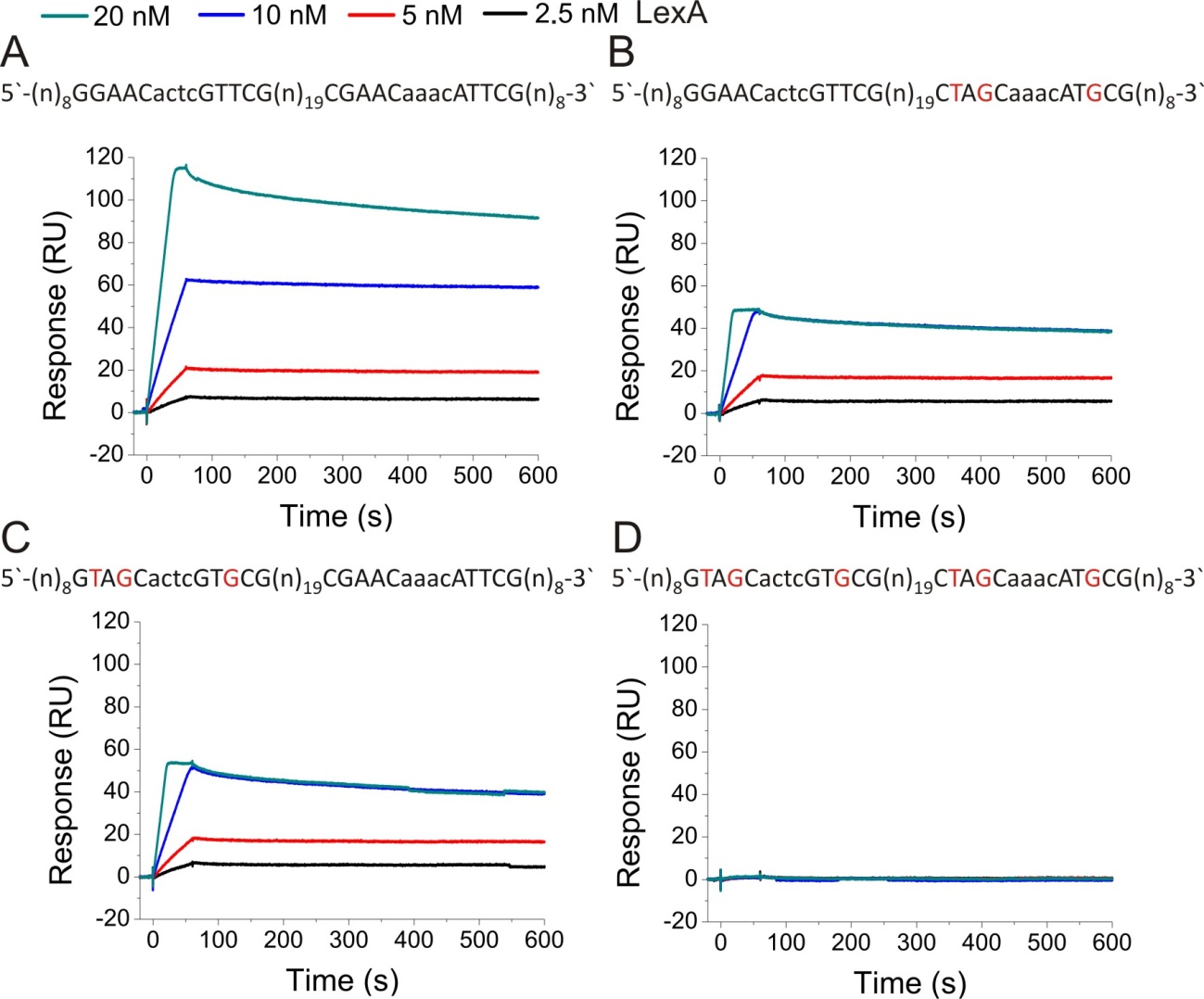


**Supplementary Figure S1.** SPR sensorgrams of the *B. thuringiensis* LexA interaction with the wild-type *P3* promoter (**A**) and mutant derivatives (**B-D**). LexA was injected over each immobilized DNA fragment (~ 60 RU) for 60 s at a flow rate of 100 µl/min. Operator sequences are presented above each graph, with bases that are conserved in LexA binding sites in capital letters and base substitutions shown in red. The sensorgrams shown are representative experiments, which were performed in duplicate.


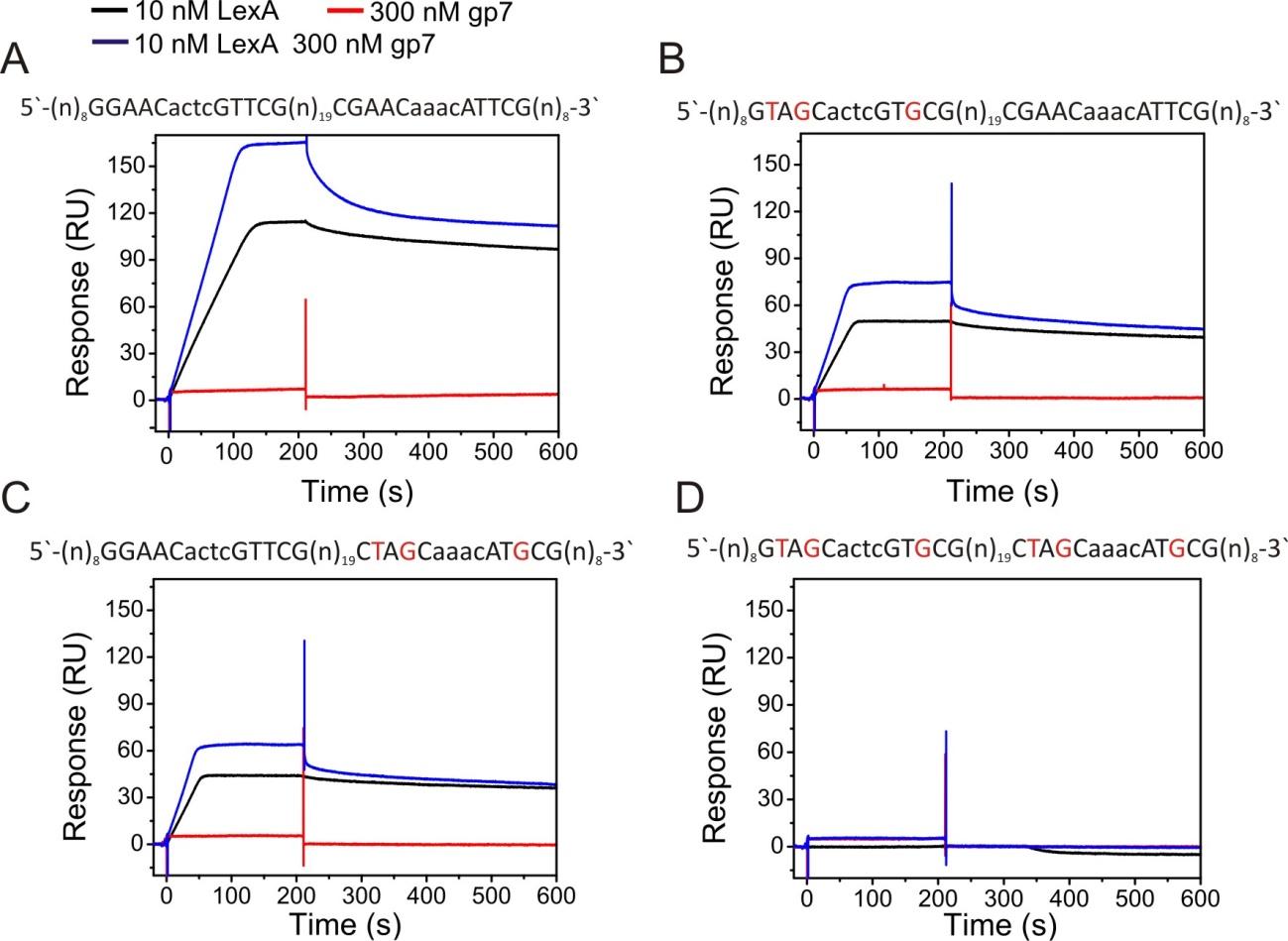


**Supplementary Figure S2.** SPR sensorgrams of the *B. thuringiensis* LexA and GIL01 gp7 interaction with a 63 bp wild-type *P3* promoter fragment (**A**) and various mutant derivatives (**B-D**). Proteins were injected over each immobilized DNA fragment (~ 65 RU) for 210 s at a flow rate of 100 µl/min. The operator sequences are presented above each graph, with the bases that are conserved in LexA binding sites in capital letters and base substitutions shown in red. The protein concentrations used are denoted above each sensorgram. The sensorgrams shown are representative experiments, which were performed in duplicate.


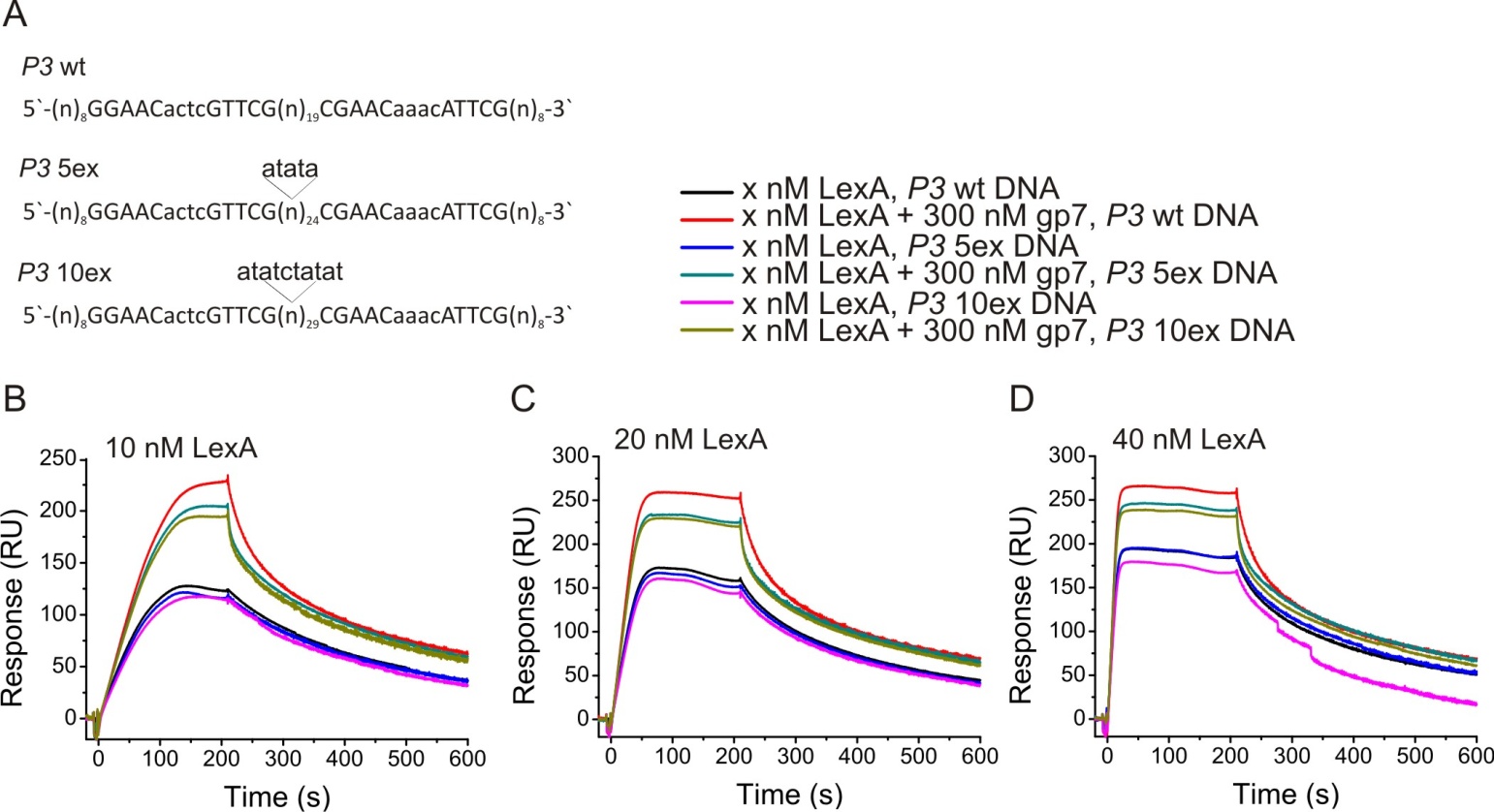


**Supplementary Figure S3.** (**A**) The panel shows the operator sequences used in this SPR analysis. Probes *P3* 5ex and 10ex contain an additional 5 and 10 nucleotides, respectively, inserted between the two SOS boxes. (**B-D**) SPR sensorgrams of the *B. thuringiensis* LexA and GIL01 gp7 interaction with immobilized wild-type *P3* promoter fragment and various mutant derivatives. Proteins were injected over each immobilized DNA fragment (~ 65 RU) for 210 s at a flow rate of 100 µl/min. The sensorgrams shown are representative experiments and all were performed in duplicate.


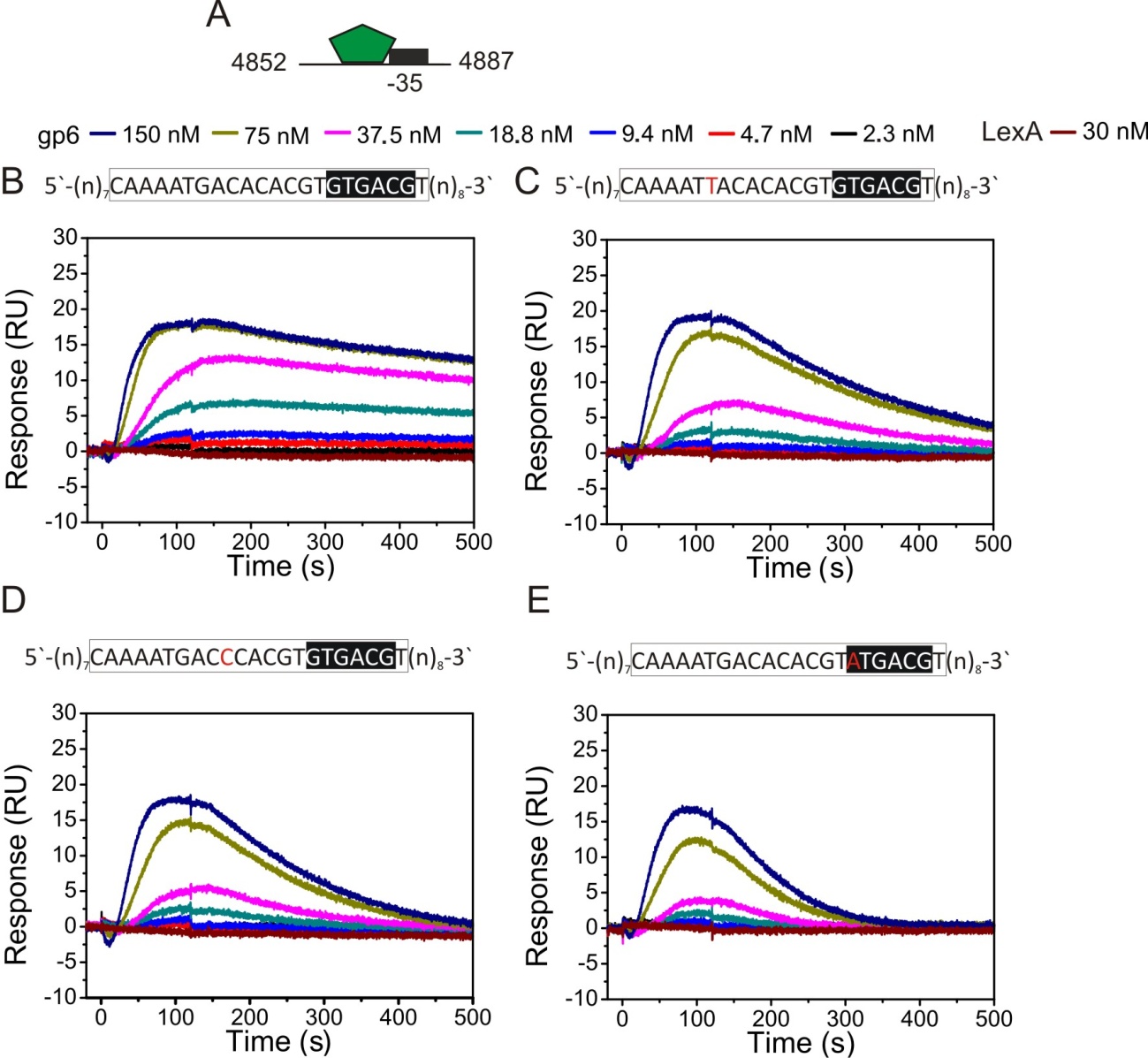


**Supplementary Figure S4.** (**A**) Schematic representation of the *P3* promoter fragment immobilized on the streptavidin chip. The green pentagon shows gp6 binding to the DNA, the -35 promoter element is represented as a black box and the GIL01 genome sequence coordinates of the probe are indicated. SPR sensorgrams are also shown of the *B. thuringiensis* LexA and GIL01 gp6 interaction with the wild-type gp6 binding site (**B**) and various mutant derivatives (**C-E**). The sequence of each probe is shown above each graph, with the gp6-binding sequence, as determined by DNaseI footprint assays, being boxed. The *P3* -35 promoter element is shown in black and substitutions are in red. Proteins were injected over each immobilized DNA fragment (~ 30 RU) for 120 s at a flow rate of 100 µl/min. The sensorgrams shown are representative experiments, which were performed in duplicate.

**gp7** 1 MRDKLLDFIIELSQSSKQVVSKSYVIDRLMQVTKEDYKELEKNVEGKKDD-------- *Bacillus thuringiensis* phage GIL01
NP_943753.1 1 MRDKLLDFIIELSQSSKQVVSKSYVIDRLMQVTKEDYKELEKNVEGKKDD-------- *Bacillus thuringiensis* phage Bam35c
EOQ01043.1 1 MRDKLLDFIIELSQSSTQVVSKKYVIDRLMQVTKEDFKELEKNAEEKKK--------- *Bacillus cereus* VD184
AFX65214.1 1 ----MLDFIIELSQSSTQVVSKKYVIDRLMQVTKEDFKELEKNAEEKKK--------- *Bacillus* *cereus* phage Sand
ETE93514.1 1 MRDKLLDFIIELSQSSKQVVSKSYVIDRLMQVTKEDYKELEKNAEGKKGEG------- *Bacillus thuringiensis* serovar aizawai
EOP13606.1 1 MRDKLLDFIIELSQSSKQVVSKSYVIDRLMQVTKEDYKELEKKTENAEKD-------- *Bacillus cereus* HuB13-1
AFX65199.1 1 MRDKLLDFIIELSQSSKQVVAKDYVIDRLMQVTKEDYKEMEKKTEGKKN--------- *Bacillus cereus* phage Lima
YP_224105.1 1 MRDKLLDFIIELSQSSKQVVCHRQVNASNKRRLQGTRKENRKCGKGLAKLLVFFICPK *Bacillus thuringiensis* phage GIL16c
YP_002302519.1 1 MRDKVLDLIIELSKSTKQVVAKDFIINELYKIAKEDESKEKETSK------------- *Bacillus* *anthracis* phage AP50
CKE78795.1 1 MRDKVLDLIIELSKSTKQVVAKDFIINELYKIAKEDEEKKMGES-------------- *Streptococcus pneumoniae*
AFX65202.1 1 MRDKLLDLIVELSKSTKQVIAKDYIIQELYVILKEDEENGTTNTK------------- *Bacillus* *cereus* phage Sato

**Supplementary Figure S5.** Amino acid sequence alignment of gp7 homologues.

*

WP_098102508.1 1 ---MLTDREKEALSCIRGYMRQNGFAPSVREMAGLLFVSHKTAHRYMIQLETKGHIKRIHHRSRAIQLCV *Bacillus wiedmannii*
WP_050224777.1 1 ---MLTDREKEALACIRVYMRQNGFAPSVREMAGLLFVSHKTAHRYMIQLETKGHIKRIHHRSRAIQLCV *Streptococcus pneumoniae*
YP_002302518.1 1 ---MLTDREQEALACISGYMRQNGFAPSVREMAGLLFVSHKTAHRYMIQLETKGHIKRIHHRSRAIQLCV *Bacillus anthracis*
**gp6** 1 ---MLTPREQDTLECISGYMEEFGFAPSVRDMASRMYVSHKTAHRYLMQLESKGRIRRVHQRPRAIQLL- *Bacillus thuringiensis*
EAO54930.1 1 MTKMLTPREQDTLECISGYMEEFGFAPSVRDMASRMYVSHKTAHRYLMQLESKGRIRRVHQRPRAIQLL- *Bacillus thuringiensis*
WP_000957403.1 1 ---MLTPREQDTLECISGYMEEFGFAPSVRDMASRMYVSHKTAHRYLMQLESKGRIRRVHQRPRAIQLL- *Bacillus sp.*
WP_048538928.1 1 ---MLTPREQDALECISGYMEEFGFAPSVRDMASRMYVSHKTAHRYLMQLESKGRIKRLQHRPRAIQLI- *Bacillus cereus*
WP_100632412.1 1 ---MLTAREQDALECISGYMEEFGFAPSVRDMASRLYVSHKTAHRYLMQLESKGRIKRLHHRPRAIQLY- *Bacillus cereus*
WP_098096911.1 1 ---MLTEREQEAMDCIAGYMEEFGFAPSVREMASRLYVSHQTAHRYISQLESKGRIQRIHHRPRAIRLLI *Bacillus wiedmannii*
WP_098624579.1 1 ---MLTTREQDAMDCITGYMDEFGFAPSVREMASRMFVSHNMAHRYLTQLESKGCIKRMPHIPRAIQIC- *Bacillus sp. AFS075034*
WP_048533816.1 1 ---MLSEREKEVMECIQDYMTEFGFAPSVRDLGSRLYVSYQTAHRYLLQLETKGKIKRSHHKSRSIQIC- *Bacillus cereus*
WP_000949573.1 1 ---MLSKREEEVMECIEDYMIEFGFAPSVRDLGSRLYVSYQTAHRYLLQLETKGKIKRSHHKSRSIQIC- *Bacillus cereus*

**Supplementary Figure S6.** Amino acid sequence alignment of gp6 homologues. A red asterisk indicates the lysine (K38) that is important for gp6 binding to DNA.

gp6 site -35 *dinBox*2 -10

NC_011523.1 1 GTCCAAAATGACACATTTGTGACGTTACAAG**CGAAC**ATAT**GTTTG**GGTTATACTTGTCAC
NC_004721.2 1 GTCCAAAATGACACACGTGTGACGTT-ACAC**AGAAC**AATC**GTTCG**TATTATAGTAGATTT
KC152967.1 1 TGCAAAAATGTGACAATTGTCACGTTACACA**GGAAC**GTCC**GTTCG**TATTATAGTATACAT
CP010096.1 1 GTCCAAAATGACACACGTGTGACG-TTATGC**GGAAC**AATT**GTTCG**TATTATAGTATACAT
AY701338.1 1 GTCCAAAATGACACACGTGTGACG-TTATGC**GGAAC**AATT**GTTCG**TATTATAGTATACAT
KC152964.1 1 GTCCAAAATGACACACGTGTGACG-TTATGC**GGAAC**GTCC**GTTCG**TATTATAGTATACAT
KC152966.1 1 GTCCAAAATGACACACGTGTGACG-TTATGC**GGAAC**ACTC**GTTCG**TATTATAGTATACAT
KC152965.1 1 GTCCAAAATGACACACGTGTGACG-TTATGC**GGAAC**ACTC**GTTCG**TATTATAGTATACAT
AJ536073.2 1 GTCCAAAATGACACACGTGTGACG-TTATGC**GGAAC**ACTC**GTTCG**TATTATAGTATACAT
AY257527.1 1 GTCCAAAATGACACACGTGTGACG-TTATGC**GGAAC**ACTC**GTTCG**TATTATAGTATACAT
CP009346.1 1 GTCCAAAATGACACACGTGTGACG-TTATGC**GGAAC**ACTC**GTTCG**TATTATAGTATACAT
CP013282.1 1 GTCCAAAATGACACACGTGTGACG-TTATGC**GGAAC**ACTC**GTTCG**TATTATAGTATACAT
CP003767.1 1 GTCCAAAATGACACACGTGTGACG-TTATGC**GGAAC**ACTC**GTTCG**TATTATAGTATACAT
KC152968.1 1 GTCCAAAATGACACACGTGTGACG-TTATGC**GGAAC**ACTC**GTTCG**TATTATAGTATACAT

 *dinBox*3 RBS

NC_011523.1 61 AT------CAAGCA----AT-AAGGAGT *Bacillus* *anthracis* phage AP50
NC_004721.2 60 ATA-**AGAAC**AAAC**ATTCG**ATTAAGGAGT *Bacillus cereus* plasmid pBClin15
KC152967.1 61 ATAG**CGAAC**AAAC**ATTCG**AT-AAGGGGT *Bacillus cereus* phage Sole
CP010096.1 60 ATAC**AGAAC**AAAC**ATTCG**AT-AAGGAGT *Bacillus thuringiensis* plasmid pBMBLin15
AY701338.1 60 ATAC**AGAAC**AAAC**ATTCG**AT-AAGGAGT *Bacillus thuringiensis* phage GIL16c
KC152964.1 60 ATAG**AGAAC**AAAC**ATTCG**AT-AAGGAGT *Bacillus cereus* phage Lima
KC152966.1 60 ATAC**CAAAC**AAAC**ATTCG**AT-AAGGAGT *Bacillus cereus* phage Emet
KC152965.1 60 ATAC**CAAAC**AAAC**ATTCG**AT-AAGGAGT *Bacillus cereus* phage Sato
AJ536073.2 60 ATAG**CGAAC**AAAC**ATTCG**AT-AAGGAGT *Bacillus thuringiensis* phage pGIL01
AY257527.1 60 ATAG**CGAAC**AAAC**ATTCG**AT-AAGGAGT *Bacillus thuringiensis* phage Bam35c
CP009346.1 60 ATAG**CGAAC**AAAC**ATTCG**AT-AAGGAGT *Bacillus thuringiensis* plasmid 5
CP013282.1 60 ATAG**CGAAC**AAAC**ATTCG**AT-AAGGAGT *Bacillus sp.* phage pGIL02
CP003767.1 60 ATAG**CGAAC**AAAC**ATTCG**AT-AAGGAGT *Bacillus thuringiensis* plasmid pBTHD789-4
KC152968.1 60 ATAG**CAAAC**ATAC**ATTCG**AT-AAGGAGT *Bacillus cereus* phage Sand

**Supplementary Figure S7.** Nucleotide sequence alignment of the *P3*-like promoter regions. Boxes mark the gp6 and LexA binding sites, the promoter elements and the ribosomal binding site (RBS). The arrow indicates the *P3* transcription start site.

1. Fornelos,N., Bamford,J.K. and Mahillon,J. (2011) Phage-borne factors and host LexA regulate the lytic switch in phage GIL01. *J Bacteriol*, **193**, 6008–6019.

2. Jensen,G.B., Wilcks,A., Petersen,S.S., Damgaard,J., Baum,J.A. and Andrup,L. (1995) The genetic basis of the aggregation system in *Bacillus thuringiensis* subsp. israelensis is located on the large conjugative plasmid pXO16. *J. Bacteriol.*, **177**, 2914–7.

3. Baba,T., Ara,T., Hasegawa,M., Takai,Y., Okumura,Y., Baba,M., Datsenko,K.A., Tomita,M., Wanner,B.L. and Mori,H. (2006) Construction of *Escherichia coli* K-12 in-frame, single-gene knockout mutants: the Keio collection. *Mol Syst Biol*, **2**, 2006 0008.

4. Agaisse,H. and Lereclus,D. (1994) Structural and functional analysis of the promoter region involved in full expression of the *cry*IIIA toxin gene of *Bacillus thuringiensis*. *Mol. Microbiol.*, **13**, 97–107.

5. Fornelos,N., Butala,M., Hodnik,V., Anderluh,G., Bamford,J.K. and Salas,M. (2015) Bacteriophage GIL01 gp7 interacts with host LexA repressor to enhance DNA binding and inhibit RecA-mediated auto-cleavage. *Nucleic Acids Res*, **43**, 7315–7329.

6. Joseph,P., Fantino,J.R., Herbaud,M.L. and Denizot,F. (2001) Rapid orientated cloning in a shuttle vector allowing modulated gene expression in *Bacillus subtilis*. *FEMS Microbiol. Lett.*, **205**, 91–7.

7. Kolb,A., Kotlarz,D., Kusano,S. and Ishihama,A. (1995) Selectivity of the *Escherichia coli* RNA polymerase E sigma 38 for overlapping promoters and ability to support CRP activation. *Nucleic Acids Res*, **23**, 819–826.
